# Proteomic Analyses Reveal the Mechanism of *Dunaliella salina Ds-26-16* Gene Enhancing Salt Tolerance in *Escherichia coli*

**DOI:** 10.1371/journal.pone.0153640

**Published:** 2016-05-02

**Authors:** Yanlong Wang, Bin Hu, Shipeng Du, Shan Gao, Xiwen Chen, Defu Chen

**Affiliations:** 1 Department of Genetics and Cell Biology, College of Life Sciences, Nankai University, Tianjin, 300071, China; 2 Department of Biochemistry and Molecular Biology, College of Life Sciences, Nankai University, Tianjin, 300071, China; 3 Department of Zoology and Developmental Biology, College of Life Sciences, Nankai University, Tianjin, 300071, China; Northeast Forestry University, CHINA

## Abstract

We previously screened the novel gene *Ds-26-16* from a 4 M salt-stressed *Dunaliella salina* cDNA library and discovered that this gene conferred salt tolerance to broad-spectrum organisms, including *E*. *coli* (*Escherichia coli*), *Haematococcus pluvialis* and tobacco. To determine the mechanism of this gene conferring salt tolerance, we studied the proteome of *E*. *coli* overexpressing the full-length cDNA of *Ds-26-16* using the iTRAQ (isobaric tags for relative and absolute quantification) approach. A total of 1,610 proteins were identified, which comprised 39.4% of the whole proteome. Of the 559 differential proteins, 259 were up-regulated and 300 were down-regulated. GO (gene ontology) and KEGG (Kyoto encyclopedia of genes and genomes) enrichment analyses identified 202 major proteins, including those involved in amino acid and organic acid metabolism, energy metabolism, carbon metabolism, ROS (reactive oxygen species) scavenging, membrane proteins and ABC (ATP binding cassette) transporters, and peptidoglycan synthesis, as well as 5 up-regulated transcription factors. Our iTRAQ data suggest that *Ds-26-16* up-regulates the transcription factors in *E*. *coli* to enhance salt resistance through osmotic balance, energy metabolism, and oxidative stress protection. Changes in the proteome were also observed in *E*. *coli* overexpressing the ORF (open reading frame) of *Ds-26-16*. Furthermore, pH, nitric oxide and glycerol content analyses indicated that *Ds-26-16* overexpression increases nitric oxide content but has no effect on glycerol content, thus confirming that enhanced nitric oxide synthesis via lower intercellular pH was one of the mechanisms by which *Ds-26-16* confers salt tolerance to *E*. *coli*.

## Introduction

Soil salinization is one of the major factors that limit the growth and distribution of organisms. Currently, the worldwide saline soil area is greater than 6% of the total land area [1] and is projected to increase to 50% by the year 2050 [2]. High salinity can lead to ion imbalance and hyperosmotic and oxidative stress, which can result in plant growth retardation, wilting or death [3]. Therefore, identifying novel salt-tolerant genes and elucidating their molecular mechanisms in conferring salt tolerance will provide a basis for effective engineering strategies to enhance salt tolerance to organisms. This goal is of extraordinary significance for the improvement and utilization of salinized soil.

Plant responses to salt stress have been extensively studied using morphological, physiological and ecological methods [4]. A number of important genes related to salt stress have been cloned, and their functions have been confirmed by genetic transformation [5–8]. However, plant response to salt stress is a dynamic, multi-level and holistic process involving a large number of genes and multiple signaling and metabolic pathways. High-throughput transcriptomic and proteomic assays are powerful in identifying genes implicated in the biological processes of various organisms. Transcriptomic analysis of the rosettes and roots of *Thellungiella* showed that 125 ESTs (expressed sequence tags) and 103 unigenes might be related to salt stress and include stress proteins, antioxidant enzymes, transporters, ion homeostasis, signaling components, and transcriptional regulators [9]. Transcriptomics of the salt-tolerant wheat SR3 identified the SR3 allele of *sro1*, a gene encoding a PARP [poly (ADP-ribose) polymerase] domain protein, which underpins both seedling vigor and abiotic stress tolerance by modulating redox homeostasis and maintaining genomic stability [10]. Over-expression of Ta-*sro1* in wheat and in *Arabidopsis* enhanced the activity of NADPH (nicotinamide adenine dinucleotide phosphate) oxidase, NAD(P)H dehydrogenase, ascorbate-GSH (glutathione) cycle enzymes and GSH peroxidase cycle enzymes [10]. Proteomic assay of rice roots revealed that proteins response to salt stress is primarily observed in carbohydrate regulation, nitrogen and energy metabolism, reactive oxygen species scavenging, mRNA and protein processing, and cytoskeleton stability [11]. A recent study revealed that under salt stress conditions, the differential protein expression pattern in rice roots were shifted towards proteins involved in photosynthesis, antioxidant activity, and oxidative phosphorylation [12]. Mitochondrial proteome have revealed that enzymes involved in the detoxification of ROS (reactive oxygen species) were identified as responsive to increased salinity in both genotypes, whereas manganese superoxide dismutase, serine hydroxyl methyltransferase, aconitase, malate dehydrogenase, and *β*-cyanoalanine synthase exhibited a higher abundance in the salt-tolerant amphiploid genotype [13].

*Escherichia coli* is a single-celled organism with a comprehensive and well-characterized genetic background. It is composed of the fewest protein types and its proteome and osmotic adjustment mechanisms are well understood [14–16]. Therefore, *E*. *coli* could serve as a model to study the molecular mechanisms of salt-tolerant genes. Proteomic analysis revealed that the *irrE* gene from radiation-resistant *Deinococcus radiodurans* R1 could enhance salt resistance in *E*. *coli* by functioning as a switch to regulate stress-related proteins, glycerol-degrading enzymes, and metabolic and growth-related proteins [5]. Transcriptomic and proteomic analyses revealed that IrrE, as a global regulator, regulates the EvgSA two-component system, the GadE, GadX and PurR master regulators, and 10 transcription factors; these changes consequently improved abiotic stress tolerances in the heterologous host *E*. *coli* [17]. iTRAQ (isobaric tags for relative and absolute quantification) analysis identified that proteins involved in ROS scavenging, fatty acid synthesis and vitamin synthesis were up-regulated in ethanol-tolerant *E*. *coli* [16]. The stress protein gene *SbUSP* from *Salicornia brachiata* conferred high salt and hyper osmotic resistance to *E*. *coli* [7].

*Dunaliella salina* is one of the most halotolerant photosynthetic unicellular eukaryotic organisms. It grows well under a wide range of NaCl concentrations from 0.05 M to a highly saturated solution (approximately 5.5 M). It also has superior osmotic adjustment ability when subjected to dramatic salt changes. *D*. *salina* can tolerate a two- to three-fold increase in salinity, and even a seven-fold gradient increase [18]. Previously, we screened a novel salt-tolerant gene *Ds-26-16* from a 4 M NaCl-induced *D*. *salina* cDNA library and discovered that this gene could confer salt tolerance to broad-spectrum organisms, including *E*. *coli*, *Haematococcus pluvialis* and tobacco [19]. Bioinformatics revealed that Ds-26-16 has homology with a *Xenopus* zinc finger protein and an *Oreochromis niloticus* nucleic acid binding factor. Subcellular localization showed that Ds-26-16 localizes to either the nucleus of onion or the nucleoid area of *E*. *coli* [19]. Therefore, *Ds-26-16* is possibly a salt-stress associated trans-regulatory gene. However, it is unclear how the proteome changes upon overexpression of *Ds-26-16* in *E*. *coli*. We sought to determine the relationship between these differentially expressed proteins and salt tolerance.

iTRAQ is an isotope labeling quantitative proteomic approach developed by Applied Biosystems Incorporation (Waltham, Massachusetts, USA) in 2004. This method permits multiplex quantitation of up to eight complex protein samples in a single multiplexed analysis [20] with high sensitivity [21]. So far, this technique has been extensively implemented in the field of quantitative proteomics. In this study, we employed iTRAQ to study the proteome of *E*. *coli* overexpressing *Ds-26-16* and to investigate the molecular mechanisms of Ds-26-16 that conferred salt tolerance to the bacterium. The study provides a basis for elucidating the broad-spectrum salt tolerance of this gene.

## Materials and Methods

### *E*. *coli* strains and culture conditions

Two engineered *E*. *coli* strains (p21-cDNA and p21-ORF) and a control strain pET-21b(+) [19] were used in this study. The p21-cDNA is a BL21 strain (DE3) that expresses the full length cDNA of *Ds-26-16* in pET-21b(+), whereas p21-ORF expresses the ORF (open reading frame) of *Ds-26-16* in pET-21b(+). These strains were cultivated in Luria-Bertani (LB) liquid medium containing 100 μg mL^-1^ ampicillin on a shaker at 37°C for 8 h. The cultures were inoculated 1:10 (*v*/*v*) into 27 mL 2×TY liquid medium containing 0.51 M NaCl. When *A*_600_ reached 0.6–0.8, IPTG (isopropyl *β*-D-1-thiogalactopyranoside, final concentration 1 mM) was added to the medium. After overnight induction, cell cultures were harvested for protein extraction.

### Protein extraction and iTRAQ analysis

The *E*. *coli* cell cultures were harvested by centrifugation, and the pellets were resuspended in 4 mL PBS buffer including 50 μg mL^-1^ of lysozyme. After incubation at room temperature for 30 min, the bacteria were then subjected to ultrasonic lysis (80 W, pulse duration of 1 min on-time and 1 min off-time). When the solution became clear, the samples were centrifuged at 14,800 *g* for 15 min. The resulting supernatants were used as the crude/total protein samples and stored at -80°C.

iTRAQ analysis was carried out by PTM Biolabs Inc. (Hangzhou, China). For each sample, 100 μg of protein solution was digested with trypsin (1:20, *w*/*w*) at 37°C overnight. The resulting peptides were then dried by vacuum centrifugation, resuspended in 0.5 M EDTA, and labeled using the 6-plex TMT kit (Thermo Scientific, San Jose, CA). Peptides from pET-21b(+), p21-cDNA, p21-ORF were labeled with TMT-129, TMT-130, and TMT-131, respectively. Afterwards, the labeled peptides were fractionated by SCX (strong cation exchange) chromatography, desalinated using a C18 spin column (Thermo Fisher Scientific, San Jose, USA) and freeze-dried. Then, the peptides were redissolved in buffer A [25 mM NaH_2_PO4 in 25% CAN (cerium ammonium nitrate), pH 2.7], loaded onto a 4.6×250 mm Ultremex SCX column (Thermo Fisher Scientific, San Jose, USA), and eluted at 1 mL min^-1^ with a gradient of buffer A for 10 min, 5–35% of buffer B (25 mM NaH_2_PO_4_, 1 M KCl in 25% CAN, pH 2.7) for 11 min, and 35–50% of buffer B for 1 min. Elutions were collected every minute and monitored by measuring the absorbance at 214 nm, desalted with a StrataX C18 column (Phenomenex, California, USA) and vacuum-dried. The collected peptides were eluted again with buffer A (2% acetonitrile, 0.1% formic acid) for 4 min at 8 μL min^-1^ followed by buffer B (95% CAN, 0.1% folic acid) for 40 min at 0.3 μL min^-1^ using the Shimadzu LC-20AD nano HPLC (high performance liquid chromatography) system (Shimadzu, Tokyo, Japan). After coupling Q Exactive Tandem Mass Spectrometry (MS/MS) (Thermo Fisher Scientific, San Jose, USA) with HPLC, the complete peptides were detected in an orbit trap with a resolution of 70,000. After high-energy collision dissociation with a collision energy of 27.0 and normalized collision energy strengthening to 12.0%, the fragment peptides were analyzed with MS/MS in an orbit trap with a resolution of 17,500. The electro spray voltage was 1.6 kV, and the automatic gain control was used to optimize the track spectrum. The range of the MS m/z scan was between 350 Da and 2,000 Da. Peptide and protein identification was performed using the Mascot search engine (ver. 2.3.0, Matrix Science).

### Bioinformatics analysis

The abundance of proteins identified from iTRAQ was compared between p21-cDNA and the control. A 1.5-fold cutoff [12] was set to determine up-regulated and down-regulated proteins with a *P*-value < 0.01. GO (gene ontology) annotation was conducted using the UniProt-GOA database (http://www.ebi.ac.uk/GOA/), and KEGG (Kyoto encyclopedia of genes and genomes) pathway enrichment was performed using the KEGG database (www.genome.jp/kegg/tool/map_pathway2.html). If the *P*-value < 0.05, either the GO term or KEGG pathway were considered to be significantly enriched. Proteins involved in either the enriched GO term or the KEGG pathway were considered to be major proteins involved in the salt stress signal network in *E*. *coli*. The same methods were used to analyze the proteome of p21-ORF.

### Cell density, pH, nitric oxide and glycerol content analysis

*E*. *coli* strains were grown in LB liquid medium containing 100 μg mL^-1^ ampicillin on a shaker at 37°C. When *A*_600_ reached 0.3, IPTG (final concentration 1 mM) was added. After 1.5 h, the cell cultures were centrifuged at 14,800 *g* for 3 min. Approximately 4×10^8^ cells were transferred to 2×TY medium (1 mM IPTG) with varying NaCl concentrations (0 M, 0.26 M, 0.51 M). After shaking at 37°C for 0 h, 0.5 h, 1 h, 2 h, 4 h or 8 h, the cell density was measured by blood-cell-counting plate. The pH of the ultrasonically lysed cells and the cultured medium was measured with a pH meter (model SP-701, Suntex Instruments Co., Ltd., Taipei, China). Nitric oxide and glycerol content were determined at 0.1 h and 8 h using a nitric oxide assay kit (Beyotime Institute of Biotechnology, Shanghai, China) and a method previously described by Zhao et al. [22], respectively.

### Statistical analysis

All the experiments concerning data comparisons were performed for three times. Statistical analyses were performed using the independent samples *t*-test (95% confidence) with IBM SPSS Statistics 11.0 (SPSS Inc., Chicago, USA). Values indicated by * or ** represented significantly difference at *P* < 0.05 or *P* < 0.01.

## Results

### Quantitative identification of *E*. *coli* proteome using iTRAQ

iTRAQ analysis of *E*. *coli* generated 163,035 MS/MS spectra, of which 41,212 spectra could be matched. When the matching error was set below 0.02 Da (S1A Fig), a total of 12,476 peptides were identified. Of these, 79% were 6–15 amino acids (S1B Fig). Ultimately, a total of 1,610 proteins were identified (S1 Table), accounting for 39.4% of the entire proteome (4,086) retrieved from UniProtKB. Proteins with a low molecular weight (less than 10 kDa) were also detected, of which 69.6% were 20–70 kDa (S1C Fig). Compared to the control strains, a total of 559 differential proteins were identified in the p21-cDNA strain, of which 259 were up-regulated and 340 were down-regulated (S2 Table).

### GO and KEGG enrichment analysis of the differential proteins in p21-cDNA strain

To understand the function and feature of the identified proteins, the differentially expressed proteins were annotated according to cellular component, molecular function and biological process. Of the 559 differential proteins, 490 were annotated (S2 Table). Structurally relevant proteins were localized in the cell, membrane and macromolecular complex, accounting for 50%, 30% and 13% of this subset, respectively. Molecularly functional proteins classified into catalytic activity, binding and transporter activity, accounting for 50%, 37% and 9%, respectively. Biological proteins relating to cellular processes and metabolic processes, accounting for 36% and 37%, respectively (S3 Table).

GO enrichment of biological processes was conducted with Fisher’s exact *P*-value. Eleven biological processes were identified with *P* < 0.05 as the standard (Table 1). Of these, amino acid metabolism, carbohydrate metabolism, ROS metabolism, single-organism membrane organization, transport, and oxidative phosphorylation of organic compounds have been reported to be involved in salt tolerance [9, 11, 23].

**Table 1:** GO enrichment of biological process of differential proteins in p21-cDNA strain.

Biological Process	GO Terms ID	Mapping	Background	*P-*value
**Amino sugar biosynthetic process**	0046349	3	3	0.024
**Cellular amino acid metabolic process**	0006520, 1901605, 1901607, 0009064, 0006541, 0006760, 0042559, 0009396, 0009065, 0006525, 0006526	59	168	0.034
**Cellular carbohydrate metabolic process**	0044262, 0016052, 0044275	21	51	0.037
**Cellular iron ion homeostasis**	0006879, 0055072	4	5	0.027
**Energy derivation by oxidation of organic compounds**	0015980	14	30	0.028
**Organic acid metabolic process**	0006082, 0043436, 0019752	80	228	0.014
**Pyrimidine nucleotide biosynthetic process**	0006221, 0006213, 0009174, 0006222, 0046049, 0009173, 0044205, 0046112	9	18	0.047
**Reactive oxygen species metabolic process**	0072593, 1901701, 0034599, 0034614	5	6	0.009
**Single-organism membrane organization**	0044802, 0061024, 0043165, 0071709, 0043163, 0044091, 0045229, 0051668, 0051205	6	10	0.039
**Small molecule catabolic process**	0044282, 0044712, 0046164, 0046174, 1901616, 0019405, 0019751, 0052646, 0006072	20	44	0.013
**Transport**	0006810,0051234, 0044765, 0051641	46	109	0.001

Of the 559 differential proteins, 472 were positioned into KEGG pathways. The enriched pathways were obtained when *P* < 0.05 (Table 2). Of these, nine were enriched from the up-regulated proteins, including ABC (ATP binding cassette) transporters and proteins involved in glyoxylate and dicarboxylate metabolism, sulfur metabolism, taurine and hypotaurine metabolism, butanoate metabolism, and tryptophan metabolism. Six were enriched from the down-regulated proteins, including oxidative phosphorylation, and ribosomal and peptidoglycan biosynthesis. Of these affected processes, oxidative phosphorylation, peptidoglycan biosynthesis and ABC transporters have been reported to relate to salt tolerance [12, 24, 25].

**Table 2:** KEGG pathway enrichment of differential proteins in p21-cDNA strain.

Differential proteins	KEGG pathway	Mapping	Background	*P-*value
**Up-regulated**	eco00630 Glyoxylate and dicarboxylate metabolism	9	23	0.001
**Up-regulated**	eco00920 Sulfur metabolism	6	14	0.006
**Up-regulated**	eco02010 ABC transporters	19	50	0.009
**Up-regulated**	eco00430 Taurine and hypotaurine metabolism	3	5	0.018
**Up-regulated**	eco00650 Butanoate metabolism	6	19	0.030
**Up-regulated**	eco00380 Tryptophan metabolism	3	6	0.033
**Up-regulated**	eco01120 Microbial metabolism in diverse environments	26	143	0.040
**Up-regulated**	eco00627 Aminobenzoate degradation	2	3	0.048
**Up-regulated**	eco00590 Arachidonic acid metabolism	2	3	0.048
**Down-regulated**	eco00190 Oxidative phosphorylation	15	29	0.000
**Down-regulated**	eco03010 Ribosome	21	52	0.001
**Down-regulated**	eco00550 Peptidoglycan biosynthesis	7	14	0.014
**Down-regulated**	eco03070 Bacterial secretion system	6	13	0.035
**Down-regulated**	eco00633 Nitrotoluene degradation	4	7	0.037
**Down-regulated**	eco00600 Sphingolipid metabolism	2	2	0.043

### Analysis of specific differential proteins to salt tolerance

To elucidate the mechanism of *Ds-26-16* conferred salt tolerance in *E*. *coli*, we investigated the differential proteins that strongly enriched in the above biological processes and KEGG pathways. A total of 202 main proteins were obtained (S4 Table). Of these, proteins associated with membrane proteins and ABC transporters accounted for the largest percentage (31.7%), followed by those involved in amino acid and organic acid metabolism (29.7%), carbohydrate metabolism (16.3%), energy metabolism (10.9%), oxidative stress protection (6.9%), and peptidoglycan biosynthesis (4.5%).

#### Amino acid and organic acid metabolism

Free amino acids such as proline (Pro) and glutamine (Gln) function as osmotically organic solutes under salt stress [26]. Under osmotic stress, the glutamate (Glu), Gln, aspartate (Asp), asparagine (Asn), threonine (Thr), serine (Ser), leucine (Leu) and histidine (His) concentrations were greater in the freezing-tolerant substitution wheat line compared to the sensitive line [27]. The transcription factor AsnC, which is associated with the global regulation of amino acid biosynthesis [28], was up-regulated (2.74-fold) in p21-cDNA. Proteins involved in amino acid metabolism were also differentially expressed. Of these, eleven proteins associated with Ala (alanine), Asp and Glu metabolism (S2A Fig) were altered. For instance, argininosuccinate lyase (catalyzes Asp flow into the tricarboxylic acid cycle) and aspartate carbamoyltransferase (catalyzes Asp flow into pyrimidine metabolism) were down-regulated 0.29- and 0.34-fold, respectively. The down-regulation of these proteins resulted in the accumulation of Asp. Meanwhile, asparaginase II, which catalyzes the inter-conversion between Asn and Asp, was up-regulated 1.77-fold. Similar situations were also observed in Glu/Gln-related pathways. Glutamine-fructose-6-phosphate aminotransferase (catalyzes Gln flow into amino sugar metabolism) and carbamoyl-phosphate synthase small chain (catalyzes the conversion of Gln to carbamoyl-phosphate) were down-regulated, whereas glutaminase (catalyzes the inter-conversion between Gln and Glu) was up-regulated (3.63-fold). The differential expression of these proteins increased the acidic amino acid (Asp and Glu), Asn and Gln contents to maintain the intracellular osmotic balance in p21-cDNA.

All proteins involved in arginine (Arg) biosynthesis (except glutaminase) were down-regulated (S2B Fig). Acetylglutamate kinase, acetylornithine/succinyldiaminopimelate aminotransferase, and argininosuccinate lyase were down-regulated 0.26-, 0.27-, and 0.29-fold, respectively. Meanwhile, aspartokinase, bifunctional polymyxin resistance protein ArnA, and acetylornithine/succinyldiaminopimelate aminotransferase, which are involved in the conversion of Asp into Lys, were down-regulated 0.35-, 0.23-, and 0.27-fold, respectively (S2C Fig). The down-regulation of these proteins decreased the Arg and Lys content and further increased the Asp content.

The regulation of organic acid metabolism also plays a key role in plant tolerance to saline conditions [29]. The accumulation of organic acids may serve to counter increasing cation levels (e.g., Na^+^) [30]. Three proteins involved in organic acid synthesis were up-regulated in the p21-cDNA strain (S2A Fig). Glutamate decarboxylase, which catalyzes Glu into GABA (*γ*-aminobutyric acid); 4-aminobutyrate aminotransferase, which catalyzes the inter-conversion between GABA and succinate semialdehyde; and succinate semialdehyde dehydrogenase, which catalyzes succinate semialdehyde to succinate, were up-regulated 4.11-, 2.98-, and 3.14-fold, respectively. The upregulation of these proteins resulted in an increase in GABA and succinic acid content. THF (tetrahydrofolate), a one-carbon carrier, is essential for the synthesis of purines and pyrimidines and is derived from DHF (dihydrofolate). This reaction is catalyzed by dihydrofolate reductase, whose expression is regulated in a growth-dependent manner [31]. Five proteins involved in folate biosynthesis were down-regulated (S2D Fig), whereas only dihydrofolate reductase was up-regulated (2.62-fold). The up-regulation of dihydrofolate reductase might stimulate the cell growth of the p21-cDNA strain under salt stress. Furthermore, the subunits of nitrate reductase involved in nitric oxide synthesis, including subunit 1α, subunit 2α and 2β, were up-regulated. Nitric oxide is an important signaling molecule involved in various biotic and abiotic stresses [32]. The up-regulation of nitrate reductase increased nitric oxide content to alleviate the damage caused by salt stress.

#### Energy metabolism

All 21 differential proteins (except FrdB) involved in oxidative phosphorylation were down-regulated in the p21-cDNA strain (S3A Fig). Subunits I (cyoB) and II (cyoA) of cytochrome bo terminal oxidase were down-regulated 0.35- and 0.27-fold, respectively; both subunits A (nuoA) and B (nuoB) of NADH-quinone oxidoreductase were down-regulated 0.43-fold. These changes resulted in a decrease in ATP content and increase in NADH levels. Furthermore, 5 differential proteins involved in TCA (tricarboxylic acid) cycle were all down-regulated (S3B Fig). Dihydrolipoamide acetyltransferase, 2-oxoglutarate dehydrogenase, and succinate dehydrogenase and fumarate reductase iron-sulfur protein were down-regulated 0.56-, 0.52-, and 0.54-fold, respectively. Meanwhile, transcription factor ArcA, which has been reported to repress the expression of TCA cycle genes [33] and proton translocating NADH dehydrogenase (oxidative phosphorylation) [34] in *E*. *coli*, was also up-regulated (1.55-fold), thus resulting in a decrease in TCA cycle activity and ATP levels in the p21-cDNA strain.

#### Carbohydrate metabolism

For carbohydrate metabolism, D-tagatose-1,6-bisphosphate aldolase subunit GatY, fructose bisphosphate aldolase monomer (fbaB), and transaldolase involved in the pentose phosphate pathway were up-regulated 4.35-, 3.70-, and 3.73-fold, respectively (S4A Fig), resulting in an increase in glyceraldehyde-3-phosphate, which may enter the glycolytic pathway. The enzyme 6-phosphogluconate dehydratase (edd), which is critical to this pathway, was down-regulated, thus shifting glucose-6-phosphate flow towards glycolysis. The subunit of EIIChb (ChbB) (catalyzes fructose-1,6-bisphosphate into glyceraldehyde-3-phosphate) involved in the glycolytic pathway was up-regulated (S4B Fig). The increase in glyceraldehyde-3-phosphate and glycolytic activity might cause an increase in glycerol synthesis.

**Fig 1:**
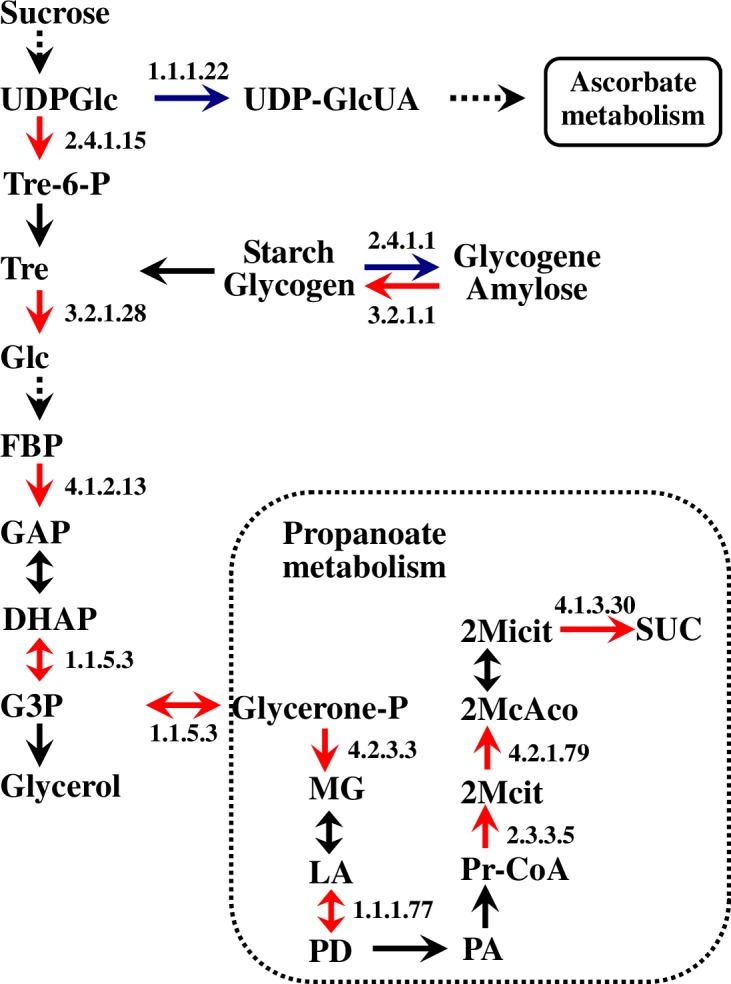
Glycerol synthesis pathway in *E*. *coli* expressing *Ds-26-16* cDNA under salt stress. Blue, down-regulated enzymes; Red, up-regulated enzymes. The number is the EC number of gene. EC: 1.1.1.22, nucleotide sugar dehydrogenase; EC: 1.1.1.77, L-1,2-propanediol oxidoreductase; EC: 1.1.5.3, glycerol-3-phosphate dehydrogenase small subunit glpA/glpB/glpC; EC: 2.3.3.5, citrate synthase; EC: 2.4.1.1, phosphorylase; EC: 2.4.1.15, a,a-trehalose-phosphate synthase (UDP-forming); EC: 3.2.1.1, glucan 1,4-a-maltohexaosidase; EC: 3.2.1.28, cytoplasmic trehalase/ periplasmic trehalase; EC: 4.1.2.13, fructose-bisphosphate aldolase; EC: 4.2.3.3, methylglyoxal synthase. Abbreviations: 2McAco, 2-Methyl-cis-aconitate; 2Mcit, 2-Methyl-citrate; 2Micit, 2-methylisocitrate; DHAP, dihydroxy acetone phosphate; FBP, fructose-1,6-bisphosphate; G3P, glycerol-3-phosphate; GAP, glyceraldehyde-3- phosphate; Glc, glucose; LA, lactaldehyde; MG, methylglyoxal; PA, propanal; PD, propanediol; Pr-CoA, propanoyl-CoA; SUC, succinate; Tre, trehalose; Tre-6-P, trehalose-6- phosphate; UDPGlc, UDP-glucose; UDP-GlcUA, UDP-D-glucuronate.

The majority of glycerol synthesis-related proteins were up-regulated in the p21-cDNA strain (Fig 1). For instance, α,α-trehalose-phosphate synthase (otsA), periplasmic trehalase, cytoplasmic trehalase, glucan 1,4-α-maltohexaosidase, fructose bisphosphate aldolase (fbaB), and glycerol-3-phosphate dehydrogenase subunits glpA, glpB, and glpC were up-regulated 4.09-, 2.21-, 6.13-, 4.17-, 3.70-, 3.20-, 3.24-, and 2.27-fold, respectively. Of these, cytoplasmic trehalase, a hypertonic response protein under aerobic conditions [14], showed the greatest increase in expression. However, nucleotide sugar dehydrogenase and phosphorylase were down-regulated, thus increasing the number of available substrates for glycerol synthesis. Interestingly, the expression of glycerol-3-phosphatase, which converts dihydroxyacetone phosphate into glycerol, was not up-regulated.

Five differential proteins involved in propanoate metabolism were all up-regulated (S4C Fig). Of these, methylglyoxal synthase, 2-methylisocitrate lyase, 2-methylcitrate dehydratase and citrate synthase were up-regulated 5.62-, 4.30-, 4.53-, and 7.08-fold, respectively. The up-regulation of these proteins shifts glycerol-3-phosphate towards propanoate metabolism and increases the succinate content (Fig 1).

#### Oxidative stress protection

As key elements in the defense system against ROS, several ROS-scavenging enzymes such as CAT (catalase), SOD (superoxide dismutase), POD (catalase-peroxidase) and osmotically inducible peroxidase OsmC were significantly up-regulated in the p21-cDNA strain (S4 Table). GSH-dependent enzymes such as GPX (glutathione peroxidase), GST (glutathione S-transferase), glutathione transferase, glutathione S-transferase domain protein, and glutaredoxin 2, all of which are vital for maintaining the redox status, were also up-regulated. In addition, the cold shock protein exoribonuclease 2 was up-regulated, as well as the transcription factor IscR, which acts as an activator in response to oxidative stress [35] (2.33-fold).

#### Membrane proteins and ABC transporters

Membrane proteins primarily include outer membrane pore proteins and outer membrane protein assembly factors (S4 Table). Of the outer membrane proteins, OmpN and OmpC were up-regulated 19.12- and 4.09-fold, respectively, whereas OmpF was down-regulated 0.38-fold. OmpF and OmpC are two of the main porins in the outer membrane of *E*. *coli*. The OmpF pore is larger and presents a higher permeability compared to OmpC [36]. OmpN displays similar functional properties (single-channel conductance) to those of OmpC [37]. An increase in OmpC expression and decrease in OmpF expression created a balance that can be reversed when growth conditions improve [37]. Lipoproteins BamA, BamB, and BamD, which comprise the BAM complex [38] and are essential for the assembly of outer membrane protein (OMP) in the outer membrane, were down-regulated 0.57-, 0.58-, and 0.58-fold, respectively, although their corresponding function in salt stress is unknown. Meanwhile, a membrane-related transcription factor BolA that is involved in cellular protection under adverse growth conditions [39] was up-regulated 2.28-fold.

ABC transporters can be distinguished into ABC exporters and ABC importers based on the polarity of transport. ABC importers, the primary transport systems in prokaryotes, actively transport various substances into cells [40]. Twenty-six ABC transporters were differentially expressed in the p21-cDNA strain (S5 Fig). The up-regulated transporters include mineral and organic ion transporters (e.g., extracellular solute-binding protein family 1 PotD, PotF), monosaccharide transporters (e.g., D-ribose transporter subunit RbsB, D-xylose ABC transporter XylF), and phosphate and acid transporters (e.g., phosphate-binding protein PstS, lysine/arginine/ornithine ABC transporter ArgT, cystine transporter FliY). These proteins primarily transport small compatible osmoprotectants into cells. The down-regulated transporters include oligosaccharide, polyol and lipid transporters (ABC transporter-related MalK, Maltose/Maltodextrin transporter MalF), and cationic amino acid transporters (periplasmic binding protein HisJ and arginine transporter subunit ArtJ). These proteins mainly transport large non-compatible substances and cationic amino acids. Meanwhile, a LYSR-type transcriptional regulator related to the transporter system [41] was also up-regulated.

#### Peptidoglycan synthesis

Peptidoglycan is the primary component of the cell wall in *E*. *coli*. Seven differential proteins involved in peptidoglycan synthesis were down-regulated in the p21-cDNA strain (S6 Fig). Of these, UDP-N-acetylenolpyruvoylglucosamine reductase (murB), UDP-N-acetylmuramoyl-tripeptide-D-alanyl-D-alanine ligase (murF), UDP-N-acetylmuramoyl-L-alanyl-D-glutamate-2,6-diaminopimelate ligase (murE), and fused penicillin-binding protein 1a (mrcA) were down-regulated 0.43-, 0.49-, 0.49-, and 0.38-fold, respectively. Glutamine-fructose-6-phosphate aminotransferase, which catalyzes Gln to glucosamine-6-phosphate and could produce the peptidoglycan precursor UDP-N-acetylglucosamine, was also down-regulated. Furthermore, the transcription factor BolA, which regulates peptidoglycan synthesis [42], was up-regulated 2.28-fold. The upregulation of BolA eventually decreased the peptidoglycan content and also, as a morphology gene, rendered the cells shorter and rounder, thus causing a decrease in surface-to-volume ratio and a reduction in the surface area exposed to the stress environment [43].

### iTRAQ analyses of p21-ORF and its comparison to that of p21-cDNA

To confirm the iTRAQ of the p21-cDNA strain, we performed iTRAQ analyses of the p21-ORF strain. A total of 542 differential proteins were identified with respect to the control strain (S2 Table). Of these, 252 were up-regulated and 290 were down-regulated. A total of 591 differential proteins were identified in either the p21-ORF or p21-cDNA strains. Of these, 509 were present in both p21-ORF and p21-cDNA, and the recurrence rate was 86.1% (509/591). The linear equation for fold change of the overlapped proteins was *F*_p21-ORF_ = 1.0268 × *F*_p21-cDNA_, R^2^ = 0.8818** (*n* = 509), suggesting that the differential proteins identified in the two strains were highly consistent. Of the remaining (82) non-overlapped proteins, 32 appeared in the p21-ORF strain. Of these, 30 showed a small change, whereas 2 were not involved in with salt tolerance. Forty-nine identified proteins appeared in the p21-cDNA strain, with 46 showing a small change and 4 not reported to be involved in salt tolerance. Only one conserved protein was down-regulated in the p21-cDNA strain but up-regulated in the p21-ORF strain (S2 Table).

Differential proteins that were either highly up- or down-regulated in the p21-cDNA strain were also greatly regulated in the p21-ORF strain (S2 Table). The membrane proteins OmpC, OmpN, outer membrane lipoprotein, OsmF, and FliY were up-regulated 4.09-, 19.12-, 7.16-, 3.99-, and 4.98-fold, respectively in the p21-cDNA strain and 5.28-, 24.95-, 9.02-, 3.86-, and 5.13-fold, respectively in the p21-ORF strain. The nitrate reductase 2 β subunit was up-regulated 2.81-fold in the p21-cDNA strain and 3.07-fold in the p21-ORF strain. The ROS scavenging proteins CAT and SOD [Cu-Zn] were up-regulated 4.07-fold and 5.13-fold, respectively, in the p21-cDNA strain and 5.02-fold and 5.99-fold, respectively, in the p21-ORF strain. Cytochrome bo terminal oxidase subunit II, which is involved in oxidative phosphorylation, was down-regulated 0.27-fold in the p21-cDNA strain and 0.20-fold in the p21-ORF strain. The transcription factors BolA and AsnC were up-regulated 2.28-fold and 2.74-fold, respectively in the p21-cDNA strain and 2.20-fold and 2.82-fold, respectively in the p21-ORF strain.

### Effect of *Ds-26-16* on cell density, pH, nitric oxide and glycerol content in *E*. *coli*

**Fig 2:**
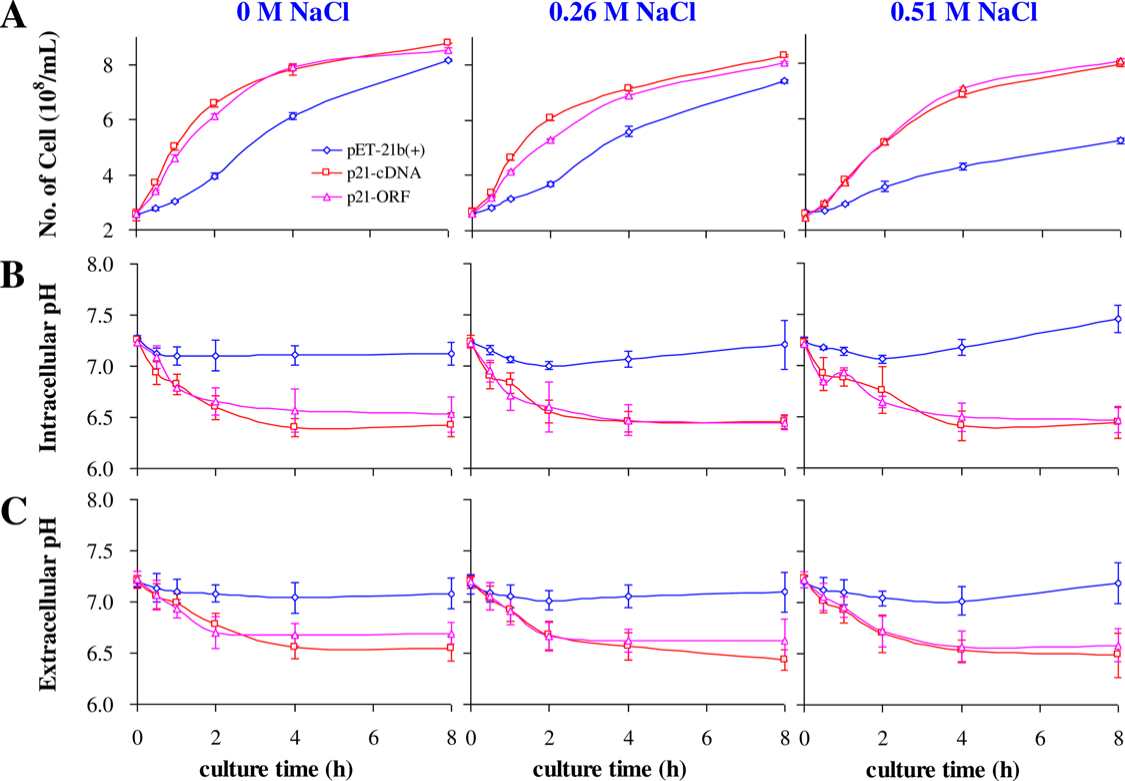
Growth status, intracellular and extracellular pH of *E*. *coli* overexpressing *Ds-26-16* under salt stress. After the cells were cultured under varying salt concentration (0 M, 0.26 M, 0.51 M) for 0 h, 0.5 h, 1 h, 2 h, 4 h or 8 h, the cell density (*A*) was measured by blood-cell-counting plate. The pH values of ultrasonically lysed cells and the cultured medium were measured as intracellular (*B*) and extracellular pH (*C*). Bars represent the averages and standard deviations from at least three independent measurements.

iTRAQ analyses of the p21-cDNA and p21-ORF strains showed that proteins associated with the synthesis of acidic amino acids, organic acids, nitric oxide, and glycerol were up-regulated. To investigate whether these changes are related to the mechanism of Ds-26-16 in salt tolerance, the intracellular and extracellular pH, nitric oxide, and glycerol contents in these two strains and a control strain were measured under salt stress. The cell densities were first determined under different NaCl concentrations (Fig 2A). Under salt-free condition, the densities of the p21-cDNA and p21-ORF strains were higher than that of the control strain at 0.5 h, 1 h, 2 h and 4 h, but they were nearly equal by 8 h. At 0.26 M NaCl, the temporal kinetics of the cell densities of these strains were almost the same as those in the salt-free conditions, except that those of the p21-cDNA and p21-ORF strains were 1.22- and 1.1-fold higher, respectively, than the control strain at 8 h. At 0.51 M NaCl, the densities of the p21-cDNA and p21-ORF strains were decreased to 90.2% and 94.7% of those of the salt-free strains at 8 h, whereas the control strain density decreased to 63.6% to that of the salt-free control strain at 8 h. These data confirm that overexpression of *Ds-26-16* confers salt tolerance to *E*. *coli*.

Under varying NaCl conditions, the intracellular pH (Fig 2B) and extracellular pH (Fig 2C) of the p21-cDNA, p21-ORF, and control strains showed a similar change pattern and stabilized after 2 h (except for the p21-cDNA strain in salt-free conditions). However, the intracellular and extracellular pH of the p21-cDNA and p21-ORF strains (6.40–7.09) were both lower than that of the control strain (7.00–7.46) at each culture time (except for 0 h), and the difference became greater with increasing salt concentration. The intracellular pH values of each strain at each culture time was slightly lower than the corresponding extracellular pH, indicating that the lowered pH derived from the alterations of the cellular metabolism. As the cell growth rate of *E*. *coli* under low pH (6.5–7.0) is higher than that under high pH (7.5–7.8) [44–45], the densities of the p21-cDNA and p21-ORF strains were higher than those of the control strain, which was probably due to a relatively lower extracellular pH in the engineered *E*. *coli*.

**Fig 3:**
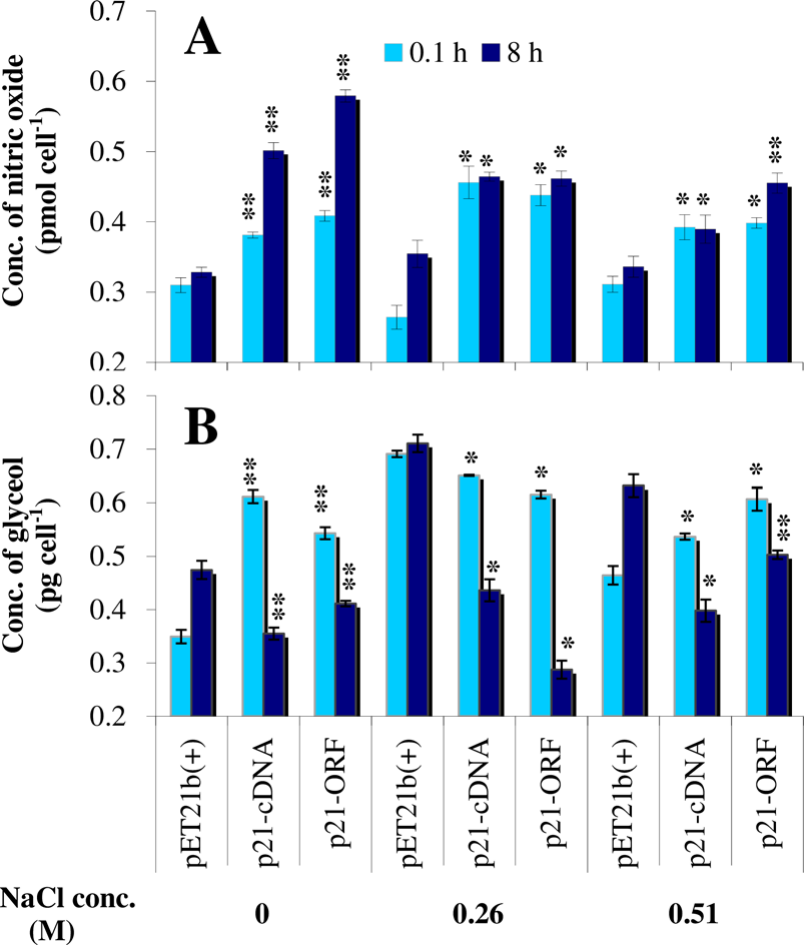
Nitric oxide and glycerol contents in *E*. *coli* overexpressing *Ds-26-16* under salt stress. After the cells were cultured under varying salt concentration (0 M, 0.26 M, 0.51 M) for 0.1 h and 8 h, (*A*) nitric oxide content and (*B*) glycerol content were determined. Bars represent the averages and standard deviations from at least three independent measurements. The difference between p21-cNDA or p21-ORF with pET-21b(+) at the same salt concentration and same culture time were compared respectively. * or ** over the bar indicates significant difference (*P* < 0.05) or highly significant difference (*P* < 0.01).

Under salt-free conditions, the nitric oxide content of the p21-cDNA and p21-ORF strains were increased 1.23- and 1.32-fold, respectively, of the value in the control strain at 0.1 h, whereas the nitric oxide content was 1.53- and 1.77-fold greater, respectively, compared to the control strain at 8 h (Fig 3A). At 0.26 M NaCl, the nitric oxide content of the p21-cDNA and p21-ORF strains was 1.73- and 1.66-fold, respectively, of the control strain at 0.1 h but was 1.30- and 1.31-fold, respectively, of the control strain at 8 h. At 0.51 M NaCl, the nitric oxide content of the engineered strains was still higher than that in the control strain, suggesting that overexpression of *Ds-26-16* increased nitric oxide synthesis and conferred salt tolerance to *E*. *coli*. The nitric oxide contents at 0.26 M and 0.51 M NaCl at 8 h were lower than those in salt-free conditions, indicating that salt stress damaged the nitric oxide synthesis pathway of engineered strains to some extent.

The glycerol content of the p21-cDNA, p21-ORF and control strains at different NaCl stresses at 0.1 h and 8 h showed no significant changes trends (Fig 3B). It appears that at 0.1 h, the glycerol content of the p21-cDNA and p21-ORF strains were clearly higher than that of the control. However, as time progressed, the content of these engineered strains at 8 h was lower than that at 0.1 h and also lower than that of the control strain at 8 h. This trend was not observed in the control strain, and no significant differences were apparent between the 8 h and 0.1 h timepoints. This result suggests that overexpression of *Ds-26-16* did not affect glycerol synthesis in *E*. *coli*.

## Discussion

As a proteomics technology, iTRAQ has higher accuracy than other non-isotope quantitative labeling techniques. It can process several samples concurrently, thus improving the throughput of comparative proteome and reducing inter-assay error to a great extent. Therefore, iTRAQ was chosen to study the proteome of an *E*. *coli* strain that overexpressed the full length of *Ds-26-16* cDNA. To confirm the reliability of the results, the proteome of an *E*. *coli* strain that overexpressed its ORF were also conducted. Possible mechanisms derived from iTRAQ analysis, including nitric oxide and glycerol synthesis, were also characterized in *E*. *coli* that overexpressed *Ds-26-16*.

**Fig 4:**
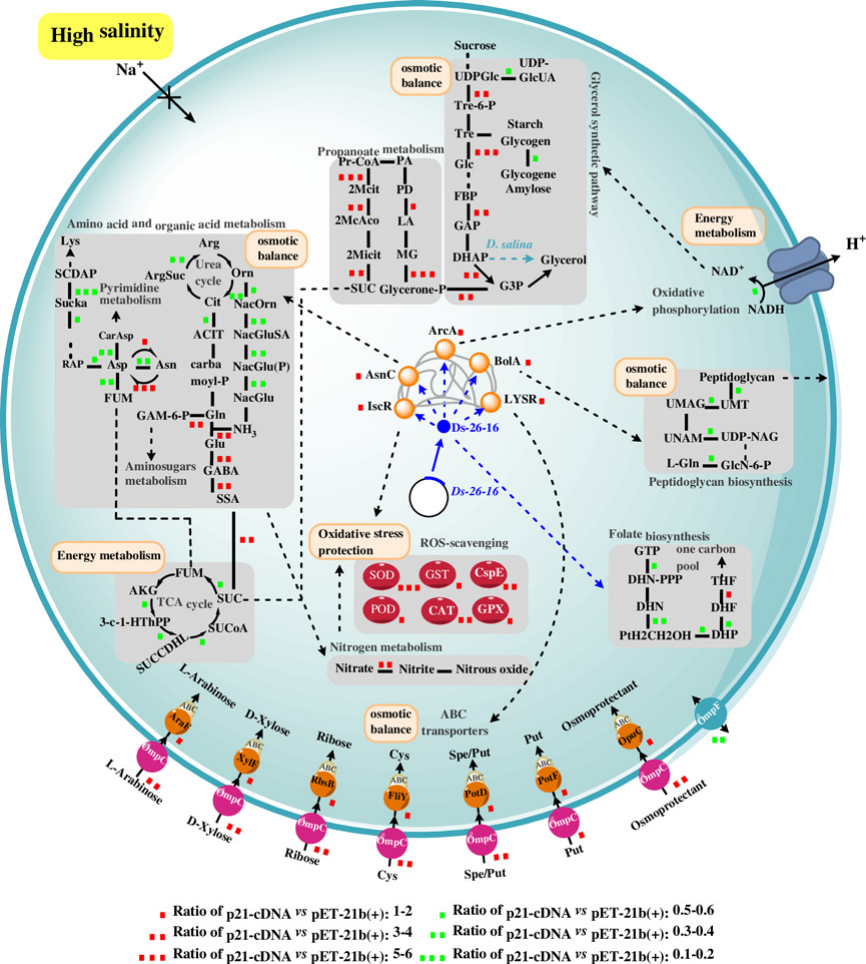
Possible mechanisms of *Ds-26-16* to enhance salt tolerance in *E*. *coli*. Abbreviations: 2McAco, 2-Methyl-cis-aconitate; 2Mcit, 2-Methyl-citrate; 2Micit, 2-methylisocitrate; 3-C-1-HThPP, 3-carboxy-1-hydroxypropyl-ThPP; ABC, ABC transporters; ACIT, N-acetyl-L-citrulline; AKG, 2-oxo-glutarate; AraF, L-arabinose transport system substrate-binding protein; ArgSuc, l-arginosuccinate; Asn, asparagine; Asp, aspartate; CarAsp, carbamoyl aspartate; CAT, catalase; Cit, citrulline; CspE, cold shock protein E; Cys, cysteate; SCDAP, N-succinyl-LL-2,6-diaminopimelate; DHAP, dihydroxy acetone phosphate; DHF, 7,8-dihydrofolate; DHN, dihydroneopterin; DHN-PPP, dihydroneopterin triphosphate; DHP, 7, 8-Dihydropteroate; FBP, fructose-1,6- bisphosphate; FliY, cystine transport system substrate-binding protein; FUM, fumarate; G3P, glycerol-3-phosphate; GAM-6-P, D-glucosamine-6-phosphate; GAP, glyceraldehyde-3-phosphate; Glc, glucose; GlcN-6-P, glucosamine 6-phosphate; GPX, glutathione peroxidase; GST, glutathione *S*-transferase; LA, lactaldehyde; Lys, Lysine; MG, methylglyoxal; NacGlu(P), N-acetylglutamate (phosphate); NacGlu, N-acetylglutamate; NacGluSA, N-acetylglutamate semialdehyde; NacOrn, N-acetylornithine; OmpC, outer membrane pore protein C; OmpF, outer membrane pore protein F; OpuC, osmoprotectant transport system substrate-binding protein; Orn, Ornithine; PA, propanal; PD, propanediol; POD, peroxidase; PotD, spermidine and putrescine transport system substrate-binding protein; PotF, putrescine transport system substrate-binding protein; Pr-CoA, propanoyl-CoA; PtH2CH2OH, 2-amino-4-hydroxy 6-hydroxymethyl-7,8-dihydropteridine; Put, putrescine; RbsB, ribose transport system substrate-binding protein; SOD, superoxide dismutase; SSA, succinate semialdehyde; SUC, succinate; SUCCDHL, S-succinyldihydrolipoamide-E; Sucka, succinylamino-oxopimelate; SUCOA, succinyl-CoA; THDPA, l-2,3,4,5-tetrahydrodipicolinate; THF, 5,6,7,8-Tetrahydrofolate; THF-L-Glu, THF-L-glutamate; THF-polyGlu, THF-L-polyglutamate; Tre, trehalose; Tre-6-P, trehalose-6-phosphate; UDPGlc, UDP-glucose; UDP-GlcUA, UDP-D-glucuronate; UDP-NAG, UDP-N-acetylglucosamine; UMAG, UDP-MurNac-L-Ala-D-Glu; UMT, UDP-MurNAc-L-Ala-D-Glu-m-DAP; UNAM, UDP-N-acetyl glucosamine acid; XylF, D-xylose transport system substrate-binding protein.

*Ds-26-16* from *D*. *salina* is a novel broad-spectrum transregulatory gene that confers salt tolerance to various organisms [19]. In this study, we showed that five transcription factors (a LYSR-type transcription factor that regulates ABC transporters; IscR, which regulates antioxidant enzymes; AsnC, which regulates amino acid biosynthesis; ArcA, which regulates energy metabolism; and BolA, which regulates membrane proteins and peptidoglycan synthesis) were up-regulated in the p21-cDNA strain that overexpressed the cDNA of *Ds-26-16*, confirming the speculation that *Ds-26-16* is a transregulatory gene. Our data suggested that Ds-26-16 may function as a global regulator by up-regulating 5 transcription factors of *E*. *coli* to regulate a number of metabolic pathways. The differential proteins identified are primarily involved in amino acid and organic acid metabolism, carbohydrate metabolism, energy metabolism, antioxidant activities, and peptidoglycan synthesis; membrane proteins and ABC transporters were also identified. Based on their function in salt tolerance, these proteins can be further classified into the following three groups: osmotic balance, energy metabolism and oxidative stress protection. Therefore, the possible mechanisms of *Ds-26-16* in enhancing salt tolerance of *E*. *coli* could be described as follows (Fig 4):

### Osmotic balance

Overexpression of *Ds-26-16* in *E*. *coli* up-regulated the transcription factor BolA, up-regulated OmpC and other outer membrane pore proteins, and down-regulated outer membrane protein assembly factors and OmpF, thus reducing the passive diffusion of osmoprotectants. Meanwhile, this overexpression also up-regulated the LYSR-type transcription factor and ABC transporters for organic ion, monosaccharide, and other small compatible osmoprotectants as well as down-regulated transporters for oligosaccharides, polyols, lipids, other large non-compatible substances, and cationic amino acids. This shift increases the compatible osmoprotectant content and inhibits the diffusion of extracellular Na^+^ into the cell. Simultaneously, Ds-26-16 overexpression up-regulated the transcription factor AsnC (which regulates the amino acid metabolism), thus increasing the levels of organic acids, acidic amino acids, Asn, and Gln; these changes were reflected by the lowered intracellular pH (Fig 2B) and extracellular pH (Fig 2C) in the engineered strains. All of these pathways eventually increased the intercellular osmotic solute content to maintain the osmotic balance under a high-salt environment.

*D*. *salina* lacks a rigid cell wall, and the cytoplasmic membrane alone makes the cell susceptible to osmotic pressure. Water flows through the cytoplasmic membrane in response to hypertonic conditions such that the original cell volume is restored [46]. However, peptidoglycan is the primary component of the cell wall in *E*. *coli*. Overexpression of *Ds-26-16* up-regulated the transcription factor BolA, which resulted in down-regulation of proteins involved in peptidoglycan synthesis. The decrease in the peptidoglycan content enhances the flexibility of the cell wall. Simultaneously, as a morphology gene, BolA renders the cells shorter and rounder, thus causing a decrease in the surface-to-volume ratio and a reduction in the surface area exposed to the stress environment [43]. Thus, overexpression of *Ds-26-16* enables *E*. *coli* cells to change their morphology to adapt to the environment similar to *D*. *salina*.

Moreover, among the enriched differential proteins, the proportion of proteins related to amino acid and organic acid metabolism, as well as membrane proteins and ABC transporters, was the greatest, indicating that regulation of osmotic balance is one of the most important mechanisms for Ds-26-16 to confer the salt tolerance to *E*. *coli*.

### Energy metabolism

Overexpression of *Ds-26-16* in *E*. *coli* up-regulated ArcA and then down-regulated TCA cycle activity and oxidative phosphorylation. It was previously reported that TCA cycle activity was decreased in the roots of salt-tolerant rice [47], although the demand for energy is commonly increased under a stress environment [23]. Therefore, a decrease in energy production may also be a mechanism of Ds-26-16 to confer salt tolerance. Meanwhile, succinic acid and GABA synthesis were also upregulated. Therefore, we speculated that overexpression of *Ds-26-16* reduced the energy demand and increased the succinic acid content and other organic acids to confer salt tolerance to *E*. *coli*.

Glycerol synthesis is an important mechanism of salt tolerance in *D*. *salina*. It was reported that a decrease in ATP content stimulated the glycerol synthesis in *D*. *salina* [48]. After expressing *Ds-26-16* in *E*. *coli*, the TCA cycle and oxidative phosphorylation were observed to be down-regulated in conjunction with up-regulation of glycerol-3-phosphate dehydrogenase and other enzymes involved in glycerol synthesis. However, glycerol-3-phosphatase was not up-regulated at the same time, which was reflected in the measurement of the glycerol content (Fig 3B). In *D*. *salina*, GPDH (glycerol-3-phosphate dehydrogenase) has two independent functional domains: a dehydrogenase and phosphorylase [6, 49]. However, only the phosphorylase domain is present in GPDH from *E*. *coli* [50]. Therefore, we proposed that *Ds-26-16* in *E*. *coli* could not substantially improve the glycerol content because of the deficiency in the phosphorylase, although most enzymes related to glycerol synthesis were up-regulated (Fig 1). As propanoate metabolism was up-regulated, the increase in glycerol-3-phosphate increased the succinate content, which is beneficial for the intercellular osmotic balance under salt stress.

### Oxidative stress protection

Overexpression of *Ds-26-16* in *E*. *coli* also up-regulated IscR followed by upregulation of active oxygen scavenging and other antioxidant enzyme activities, thus protecting the bacterium from the oxidative damage and osmotic injury caused by salt stress. Production of nitrite-dependent nitric oxide by *E*. *coli* via membrane-associated nitrate reductase also contributes to the oxidative stress protection mechanism [51]. Overexpression of *Ds-26-16* in *E*. *coli* up-regulated nitrate reductase activity and increased the nitric oxide content, which was also demonstrated by nitric oxide analysis showing that the content of the engineered strain was higher than that of the control strain regardless of applied salt stress (Fig 3A). As nitrate reductase exhibited higher activity at a lower pH [52–53], the decreased intracellular pH possibly improved the nitrate reductase activity and enhanced the nitric oxide content in the engineered strains. Thus, enhancing nitric oxide content by decreasing the intracellular pH is another mechanism by which *Ds-26-16* confers salt tolerance to *E*. *coli*.

Several major differential proteins with a large change in expression (S4 Table) could not be placed into a specific pathway although they were derived from the enrichment analysis. Some of these proteins had different expression patterns from those of a previous study. For example, ketol-acid reductoisomerase (ilvC), a cold stress-induced protein in *Bacillus subtilis* [54], was down-regulated to 0.26-fold in this study. C_4_-dicarboxylate transport gene (dctA) and cusB, which are induced by external stimuli and indole, respectively, were both down-regulated in this study in nodule bacteria [55–56]. Others had the same expression pattern as those of a previous study, although their specific role was unclear. For example, 5-methyltetrahydropteroyltriglutamate-homocysteine methyltransferase (metE), a methionine synthase that was readily inactivated in *E*. *coli* under oxidative stress [57], was down-regulated in this study (0.22-fold). 2-dehydro-3-deoxyphosphooctonate aldolase (kdsA), whose gene was down-regulated in mulberry leaves under high salt or drought stress [58], was also down-regulated in this study. The lipopolysaccharide transport periplasmic protein LptA, which is implicated in the transport of lipopolysaccharides from the inner membrane to the outer membrane of *E*. *coli* [59], was up-regulated in this study.

In summary, the proteome of *E*. *coli* that overexpress *Ds-26-16* was quantitated using iTRAQ. Our data suggest that Ds-26-16 enhances the salt tolerance of *E*. *coli* by 5 transcription factors and regulating a number of metabolic pathways, including amino acid and organic acid metabolism, energy metabolism, carbon metabolism, ROS scavenging, and peptidoglycan synthesis, as well as the membrane proteins and ABC transporters. The study provides a basis for understanding the mechanism of *Ds-26-16* in enhancing salt tolerance in *E*. *coli*. Further study regarding these mechanisms in eukaryotes such as yeast and plants is needed to elucidate the broad-spectrum salt tolerance of the gene.

## Supporting Information

S1 Fig Quantitative proteome analyses of p21-cDNA under salt stress. (*A*) Mass error distribution; (*B*) Identified peptide distribution; (*C*) Identified protein mass distribution.
(PDF)

S2 Fig Amino acid and organic acid metabolism of p21-cDNA strain under salt stress. (*A*) eco00250 Alanine, aspartate and glutamate metabolism; (*B*) eco00220 Arginine biosynthesis; (*C*) eco00300 Lysine biosynthesis; (*D*) eco00790 Folate biosynthesis.
(DOC)

S3 Fig Energy metabolism of p21-cDNA strain under salt stress. (*A*) eco00190 oxidative phosphorylation; (*B*) eco00020 cirate cycle (TCA cycle).
(DOC)

S4 Fig Carbohydrate metabolism of p21-cDNA strain under salt stress. (*A*) eco00030 pentose phosphate pathway; (*B*) eco00010 glycolysis/gluconeogenesis; (*C*) eco00640 propanoate metabolism.
(DOC)

S5 Fig ABC transporters (eco02010) of p21-cDNA strain under salt stress. (DOC)

S6 Fig Peptidoglycan biosynthesis pathway (eco00550) of p21-cDNA strain under salt stress. (DOC)

S1 Table The original protein list containing both identification and quantification information identified in p21-cDNA and p21-ORF strain using iTRAQ. (XLS)

S2 Table Differential proteins identified in p21-cDNA and p21-ORF strain under salt stress. (PDF)

S3 Table GO classification of differential proteins identified in p21-cDNA strain. (PDF)

S4 Table Main differential proteins identified in p21-cDNA strain under salt stress. (PDF)
