# Supplementary material for: Proteomic Analyses Reveal the Mechanism of Dunaliella salina Ds-26-16 Gene Enhancing Salt Tolerance in Escherichia coli
Source: PLoS One. 2016 May 2;11(5):e0153640. doi: 10.1371/journal.pone.0153640 (PMC4852897; doi:10.1371/journal.pone.0153640)
Supplement: S1 Fig — (A) Mass error distribution; (B) Identified peptide distribution; (C) Identified protein mass distribution. (PDF) [file pone.0153640.s001.pdf]

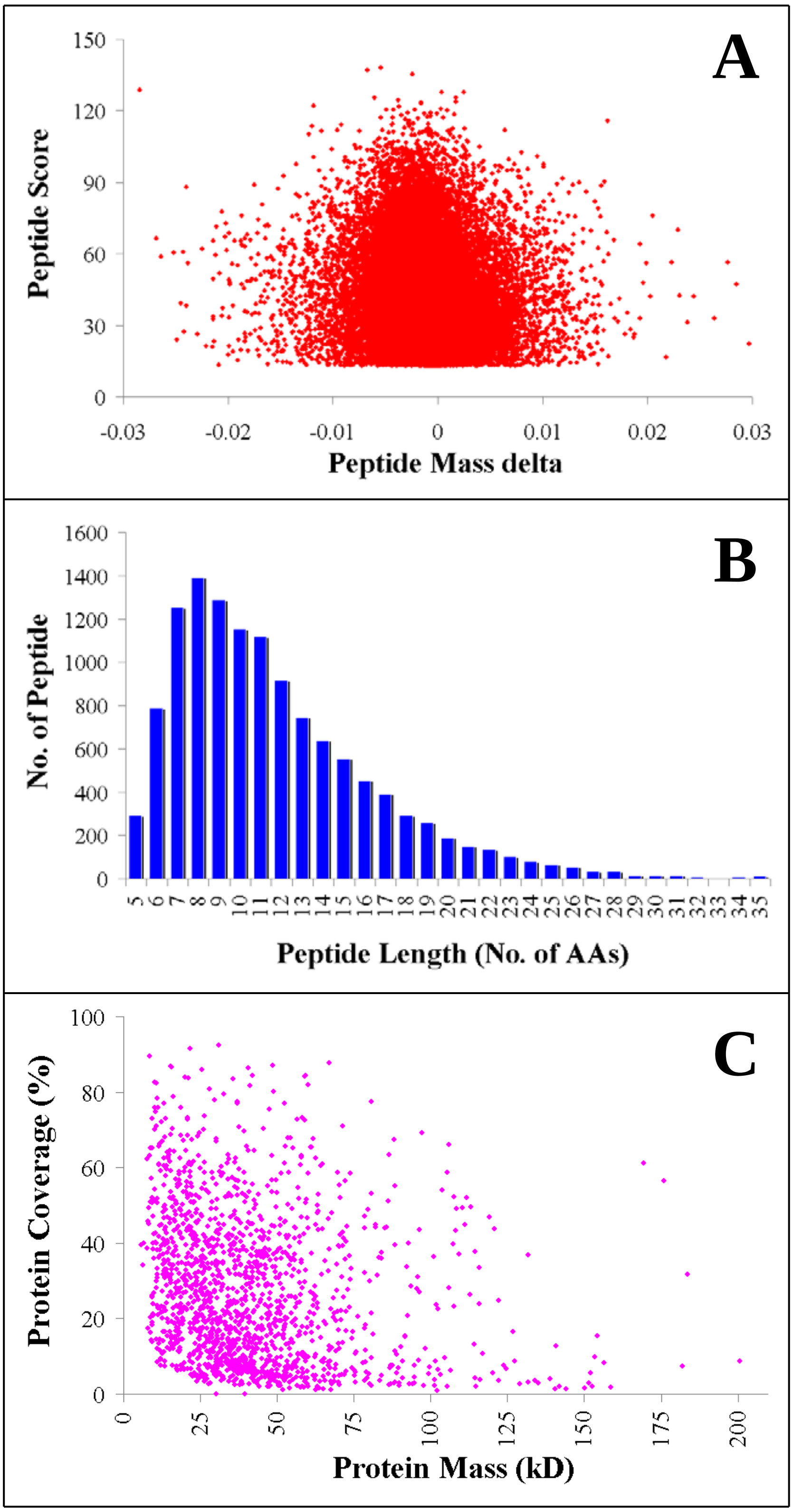

S1 Fig. Quantitative proteome analyses of p21-cDNA under salt stress. (*A*) Mass error distribution; (*B*) Identified peptide distribution; (*C*) Identified protein mass distribution
