# Supplementary material for: Proteomic Analyses Reveal the Mechanism of Dunaliella salina Ds-26-16 Gene Enhancing Salt Tolerance in Escherichia coli
Source: PLoS One. 2016 May 2;11(5):e0153640. doi: 10.1371/journal.pone.0153640 (PMC4852897; doi:10.1371/journal.pone.0153640)
Supplement: S2 Fig — (A) eco00250 Alanine, aspartate and glutamate metabolism; (B) eco00220 Arginine biosynthesis; (C) eco00300 Lysine biosynthesis; (D) eco00790 Folate biosynthesis. (DOC) [file pone.0153640.s002.doc]

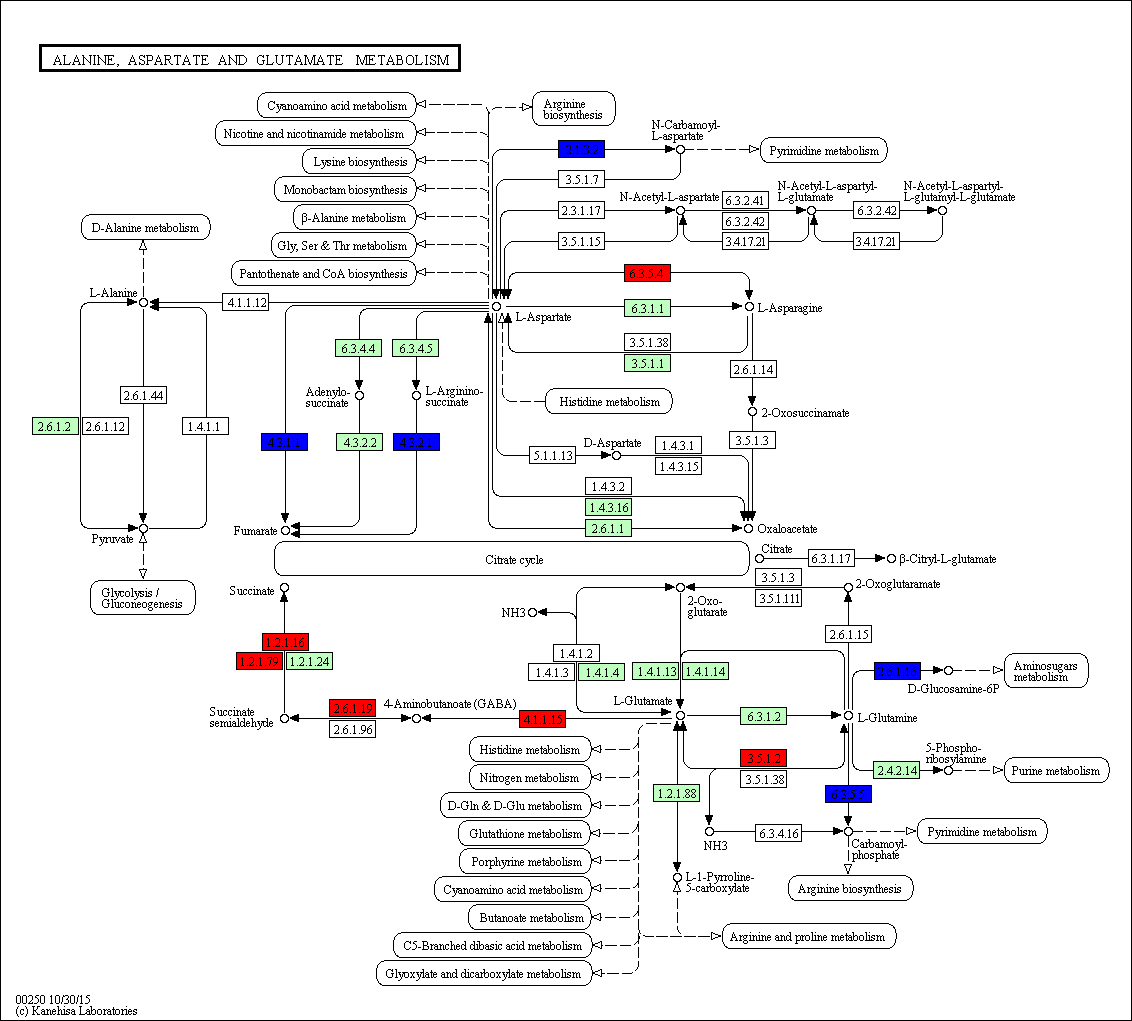

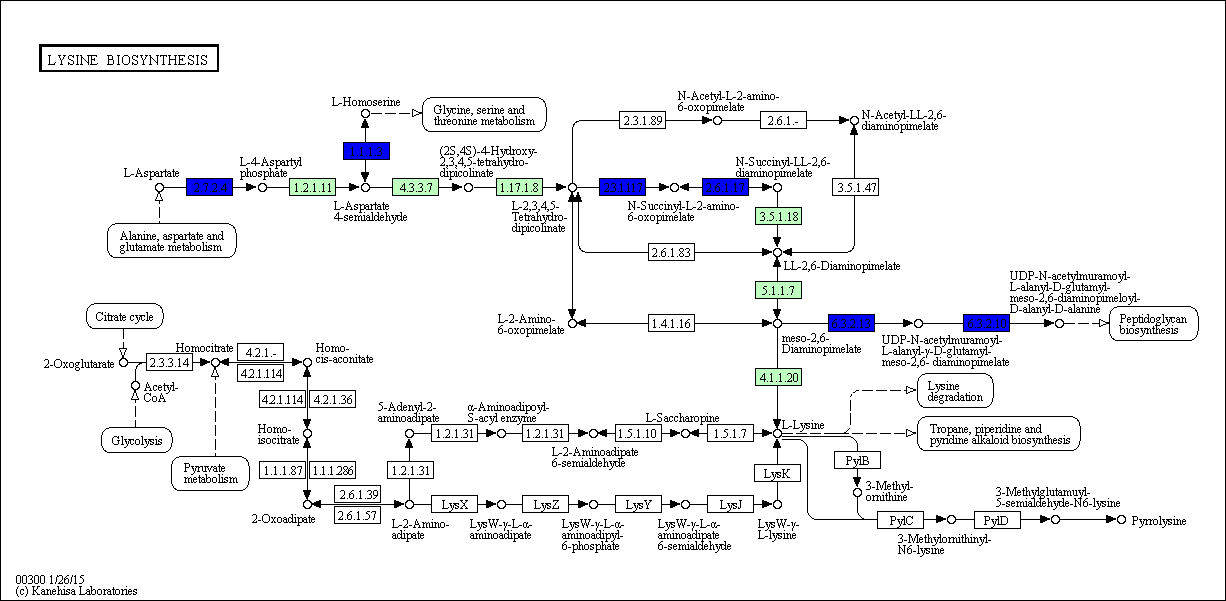


**C**


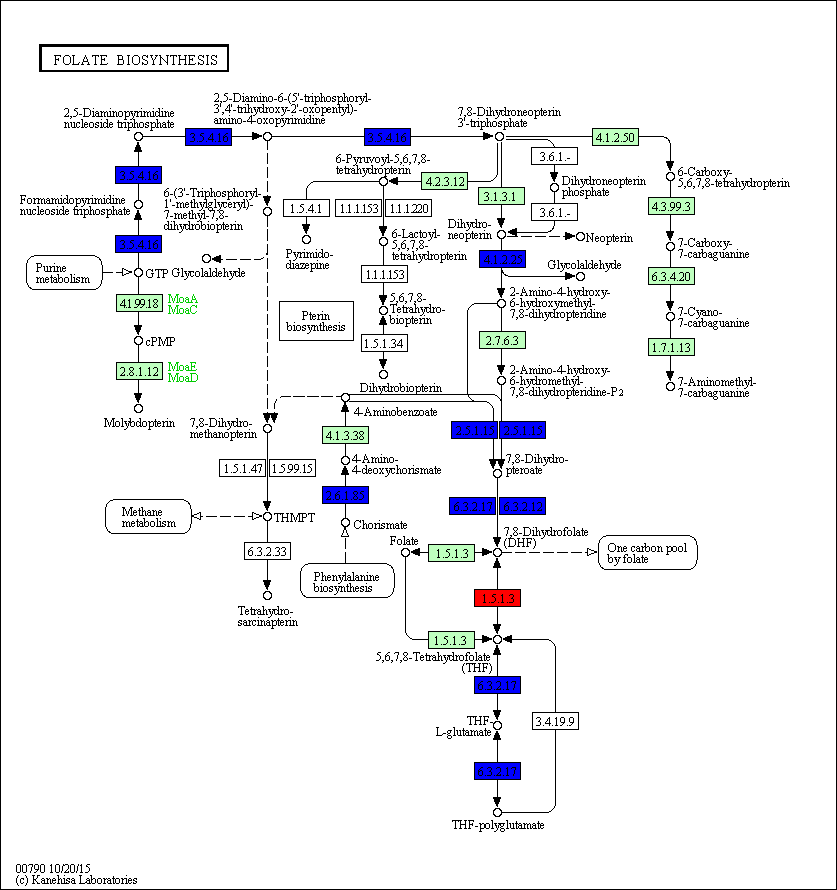

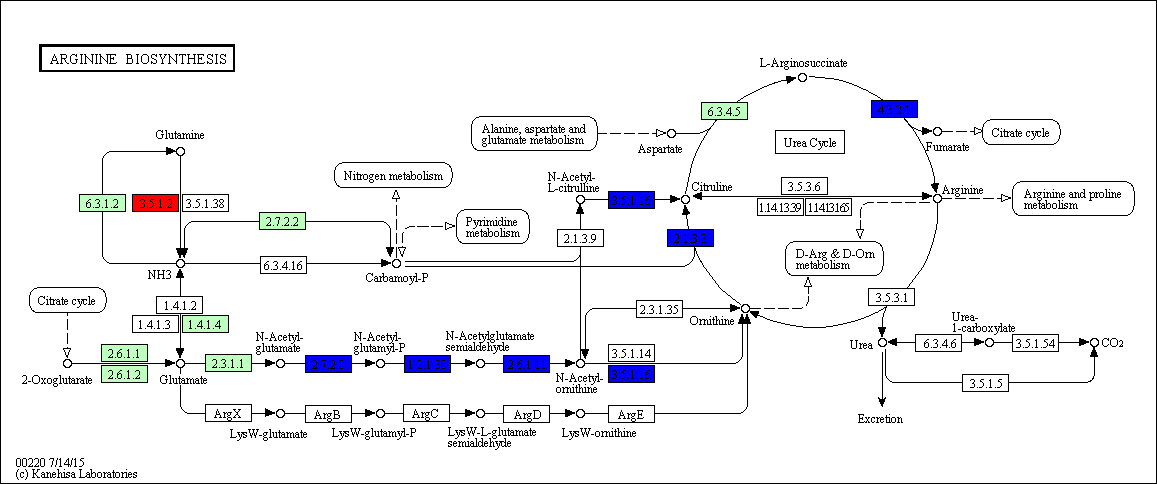


**A**

**B**

**D**

**S2 Fig. Amino acid and organic acid metabolism of p21-cDNA strain under salt stress.** (*A*) eco00250 Alanine, aspartate and glutamate metabolism; (*B*) eco00220 Arginine biosynthesis; (*C*) eco00300 Lysine biosynthesis; (*D*) eco00790 Folate biosynthesis. Blue, down-regulated enzymes; Red, up-regulated enzymes in p21b-cDNA *vs* pET-21b(+). The number is the EC number of gene. EC: 2.1.3.2, aspartate carbamoyltransferase; EC: 6.3.5.4, asparagine synthetase; EC: 4.3.1.1, aspartate ammonia-lyase; EC: 4.3.2.1, argininosuccinate lyase; EC: 1.2.1.16 1.2.1.79, Succinate semialdehyde dehydrogenase, NADP^+^-dependent; EC: 2.6.1.19, 4-aminobutyrate aminotransferase; EC: 4.1.1.15, Glutamate decarboxylase; EC: 3.5.1.2, Glutaminase; EC: 2.6.1.16, Glutamine-fructose-6-phosphate aminotransferase [isomerizing]; EC: 6.3.5.5, Carbamoyl-phosphate synthase small chain; EC: 2.7.2.8, Acetylglutamate kinase; EC: 1.2.1.38, N-acetyl-gamma-glutamyl-phosphate reductase; EC: 2.6.1.11 2.6.1.17, Acetylornithine/ succinyldiaminopimelate aminotransferase; EC: 3.5.1.16, Acetylornithine deacetylase; EC: 2.1.3.3, Ornithine carbamoyltransferase; EC: 2.7.2.4 1.1.1.3, Aspartokinase; EC: 2.3.1.117, 2,3,4,5-tetrahydropyridine-2,6-dicarboxylate N-succinyltransferase; EC: 6.3.2.13, UDP-N-acetylmuramoyl-L-alanyl-D-glutamate-2,6-diaminopimelate ligase; EC: 6.3.2.10, UDP-N-acetylmuramoyl-tripeptide-D-alanyl-D-alanine ligase; EC: 3.5.4.16, GTP cyclohydrolase 1; EC: 4.1.2.25, Bifunctional dihydroneopterin aldolase/dihydroneopterin triphosphate 2’-epimerase; EC: 2.5.1.15, 7,8-dihydropteroate synthase; EC: 6.3.1.12 6.3.2.17, Bifunctional protein FolC; EC: 1.5.1.- 1.5.1.3, Dihydrofolate reductase/dihydromonapterin reductase.
