# Supplementary material for: Proteomic Analyses Reveal the Mechanism of Dunaliella salina Ds-26-16 Gene Enhancing Salt Tolerance in Escherichia coli
Source: PLoS One. 2016 May 2;11(5):e0153640. doi: 10.1371/journal.pone.0153640 (PMC4852897; doi:10.1371/journal.pone.0153640)
Supplement: S3 Fig — (A) eco00190 oxidative phosphorylation; (B) eco00020 cirate cycle (TCA cycle). (DOC) [file pone.0153640.s003.doc]

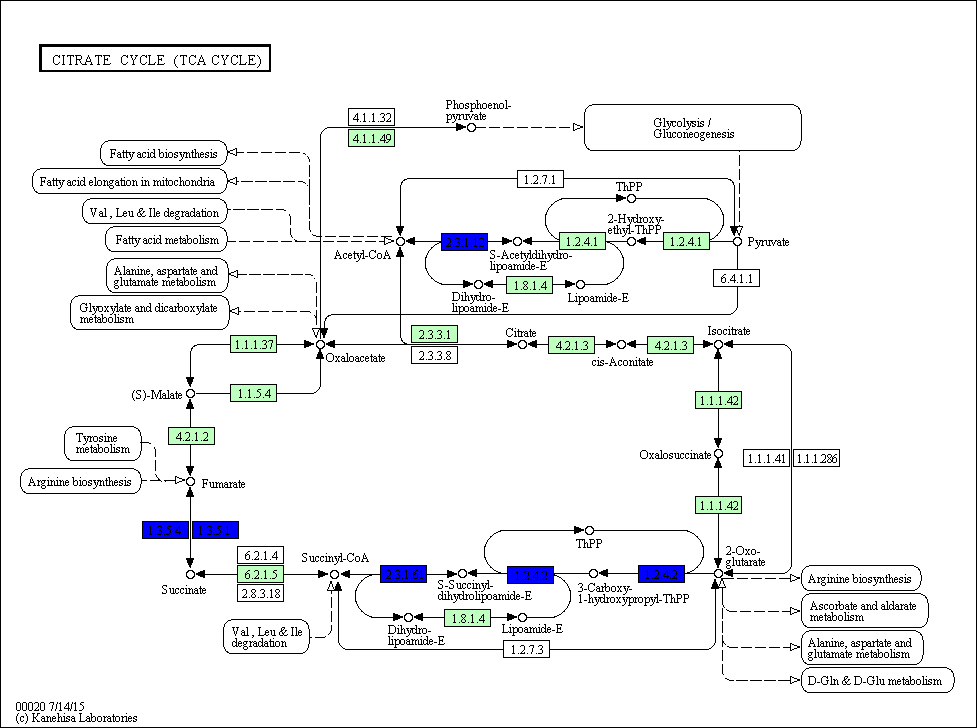

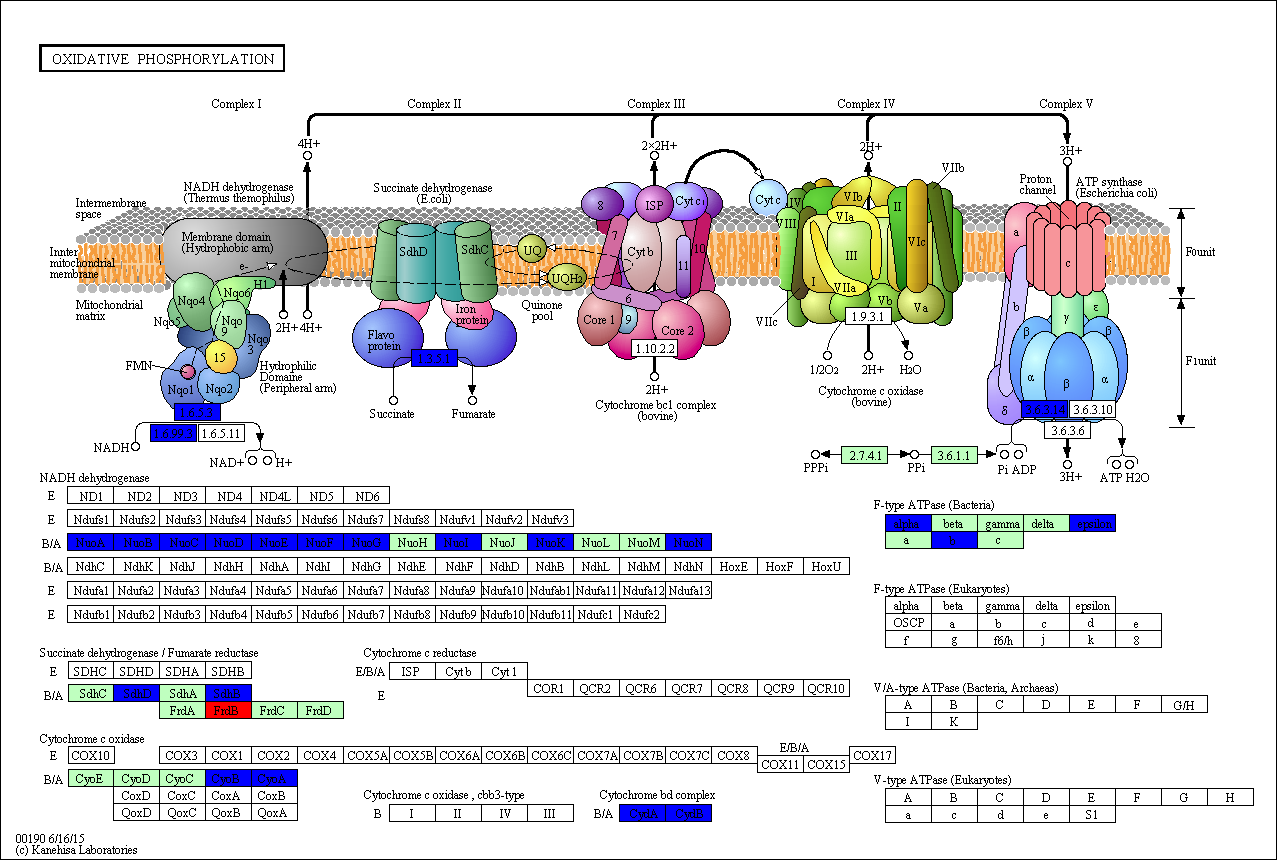


**A**

**B**

**S3 Fig. Energy metabolism of p21-cDNA strain under salt stress.** (*A*) eco00190 oxidative phosphorylation; (*B*) eco00020 cirate cycle (TCA cycle). Blue, down-regulated enzymes; Red, up-regulated enzymes in p21b-cDNA *vs* pET-21b(+).The number is the EC number of gene. EC: 1.6.5.3, NADH-quinone oxidoreductase subunit NuoA/NuoB/NuoC/NuoD/NuoE/NuoF/NuoG/NuoI/NuoK/NuoN; EC: 1.6.99.3, NADH, ubiquinone oxidoreductase II; EC: 1.3.5.1 1.3.5.4, Succinate dehydrogenase cytochrome b556 small membrane subunit (SdhD); EC: 3.6.3.14, ATP synthase subunit b; EC: 2.3.1.12, Dihydrolipoamide acetyltransferase; EC: 2.3.1.61, 2-oxoglutarate dehydrogenase, E2 subunit, dihydrolipoamide; EC: 1.2.4.2, 2-oxoglutarate dehydrogenase, E1 subunit; a, ATP synthase subunit a; epsilon, ATP synthase epsilon chain; b, ATP synthase subunit b; SdhB, Succinate dehydrogenase and fumarate reductase iron-sulfur protein; FrdB, Fumarate reductase (Anaerobic), Fe-S subunit; CyoB, Cytochrome bo terminal oxidase subunit I, subunit of cytochrome bo terminal oxidase; CyoA, Cytochrome bo terminal oxidase subunit II, subunit of cytochrome bo terminal oxidase; CydA, Cytochrome bd ubiquinol oxidase subunit I; CydB, Cytochrome bd-I terminal oxidase subunit II, subunit of cytochrome bd-I terminal oxidase.
