# Supplementary material for: Proteomic Analyses Reveal the Mechanism of Dunaliella salina Ds-26-16 Gene Enhancing Salt Tolerance in Escherichia coli
Source: PLoS One. 2016 May 2;11(5):e0153640. doi: 10.1371/journal.pone.0153640 (PMC4852897; doi:10.1371/journal.pone.0153640)
Supplement: S4 Fig — (A) eco00030 pentose phosphate pathway; (B) eco00010 glycolysis/gluconeogenesis; (C) eco00640 propanoate metabolism. (DOC) [file pone.0153640.s004.doc]

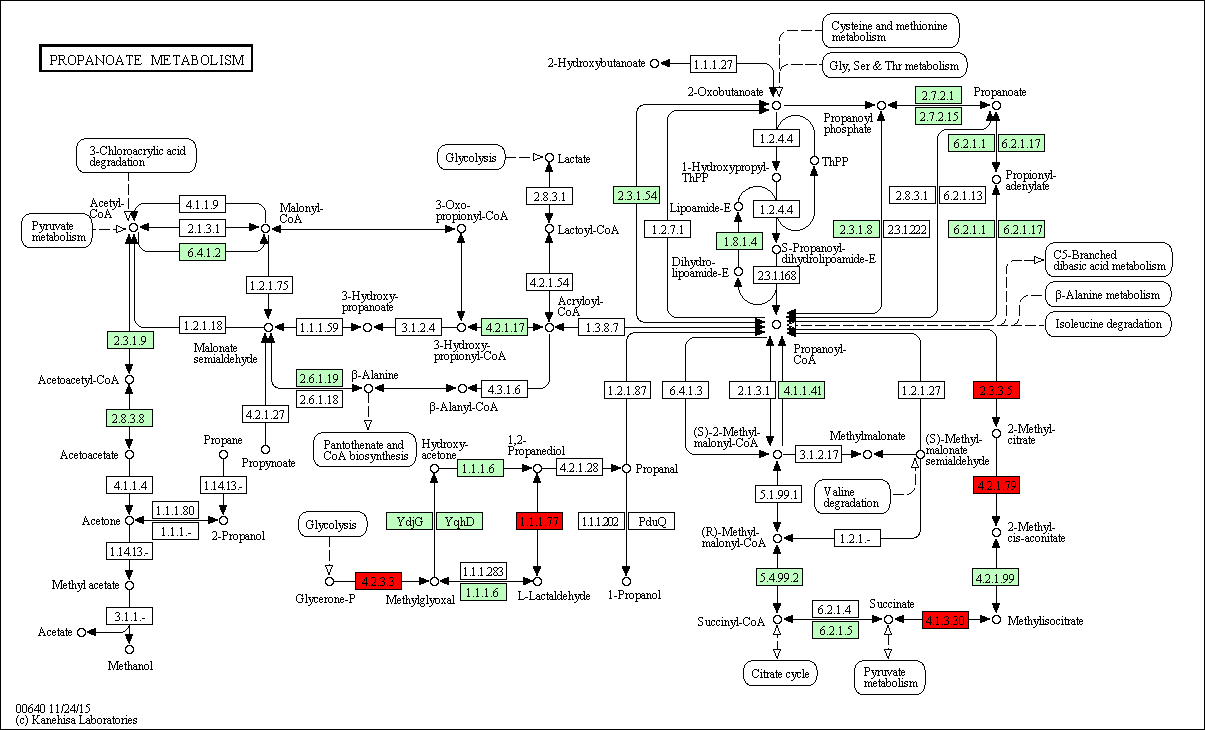

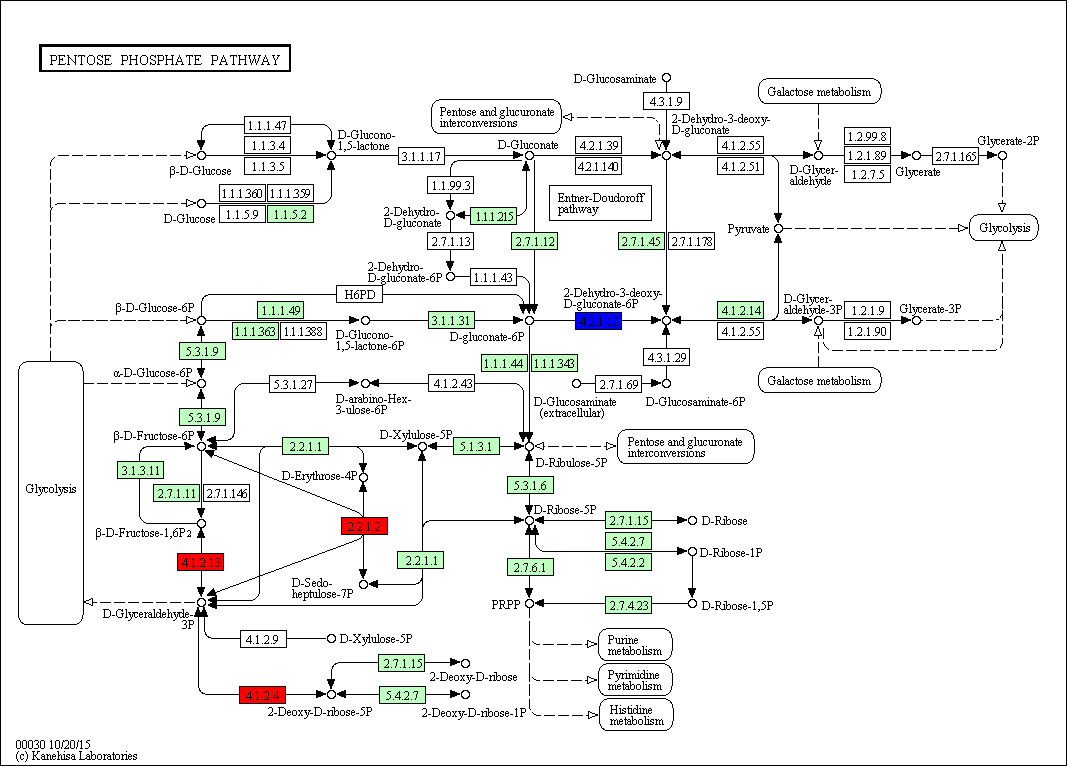


**A**


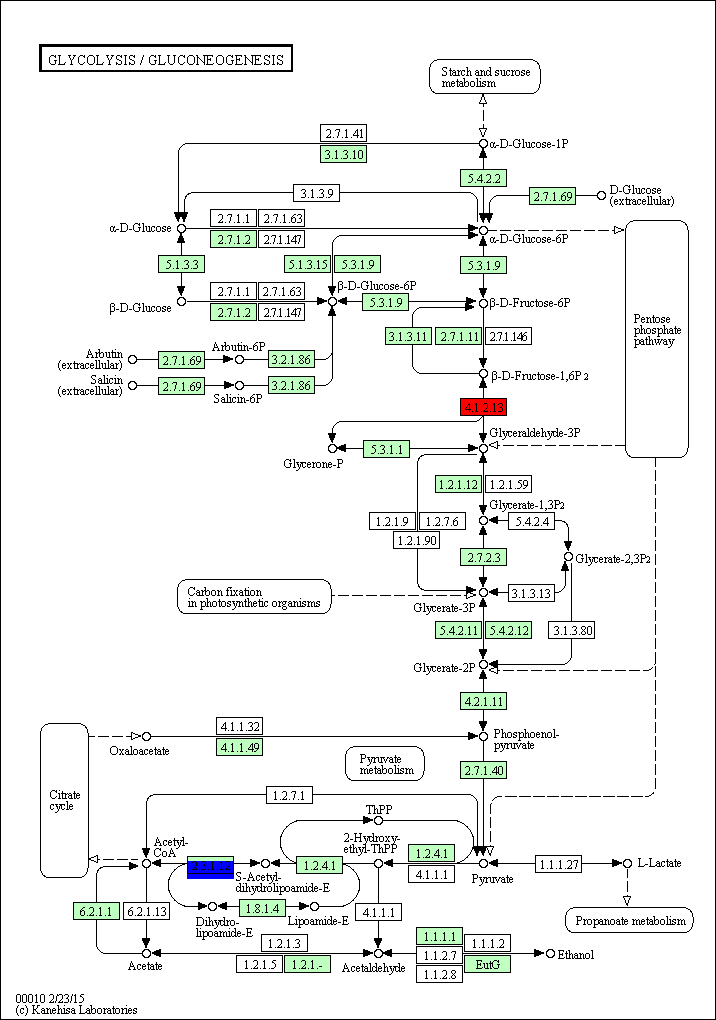


**B**

**C**

**S4 Fig. Carbohydrate metabolism of p21-cDNA strain under salt stress.** (*A*) eco00030 pentose phosphate pathway; (*B*) eco00010 glycolysis/gluconeogenesis; (*C*) eco00640 propanoate metabolism. Blue, down-regulated enzymes; Red, up-regulated enzymes in p21b-cDNA *vs* pET-21b(+). The number is the EC number of gene. EC: 1.1.1.77, L-1,2-propanediol oxidoreductase; EC: 2.2.1.2, Transaldolase; EC: 2.3.1.12, dihydrolipoamide acetyltransferase; EC: 2.3.3.5, citrate synthase; EC: 4.1.2.13, Fructose bisphosphate aldolase monomer, subunit of fructose bisphosphate aldolase class I; EC: 4.1.2.4, D-tagatose-1,6-bisphosphate aldolase subunit GatY; EC: 4.1.3.30, 2-methylisocitrate lyase; EC: 4.2.1.12, 6-phosphogluconate dehydratase; EC: 4.2.1.79, 2-methylcitrate dehydratase; EC: 4.2.3.3, methylglyoxal synthase.
