# Supplementary material for: Proteomic Analyses Reveal the Mechanism of Dunaliella salina Ds-26-16 Gene Enhancing Salt Tolerance in Escherichia coli
Source: PLoS One. 2016 May 2;11(5):e0153640. doi: 10.1371/journal.pone.0153640 (PMC4852897; doi:10.1371/journal.pone.0153640)
Supplement: S5 Fig — (DOC) [file pone.0153640.s005.doc]

**
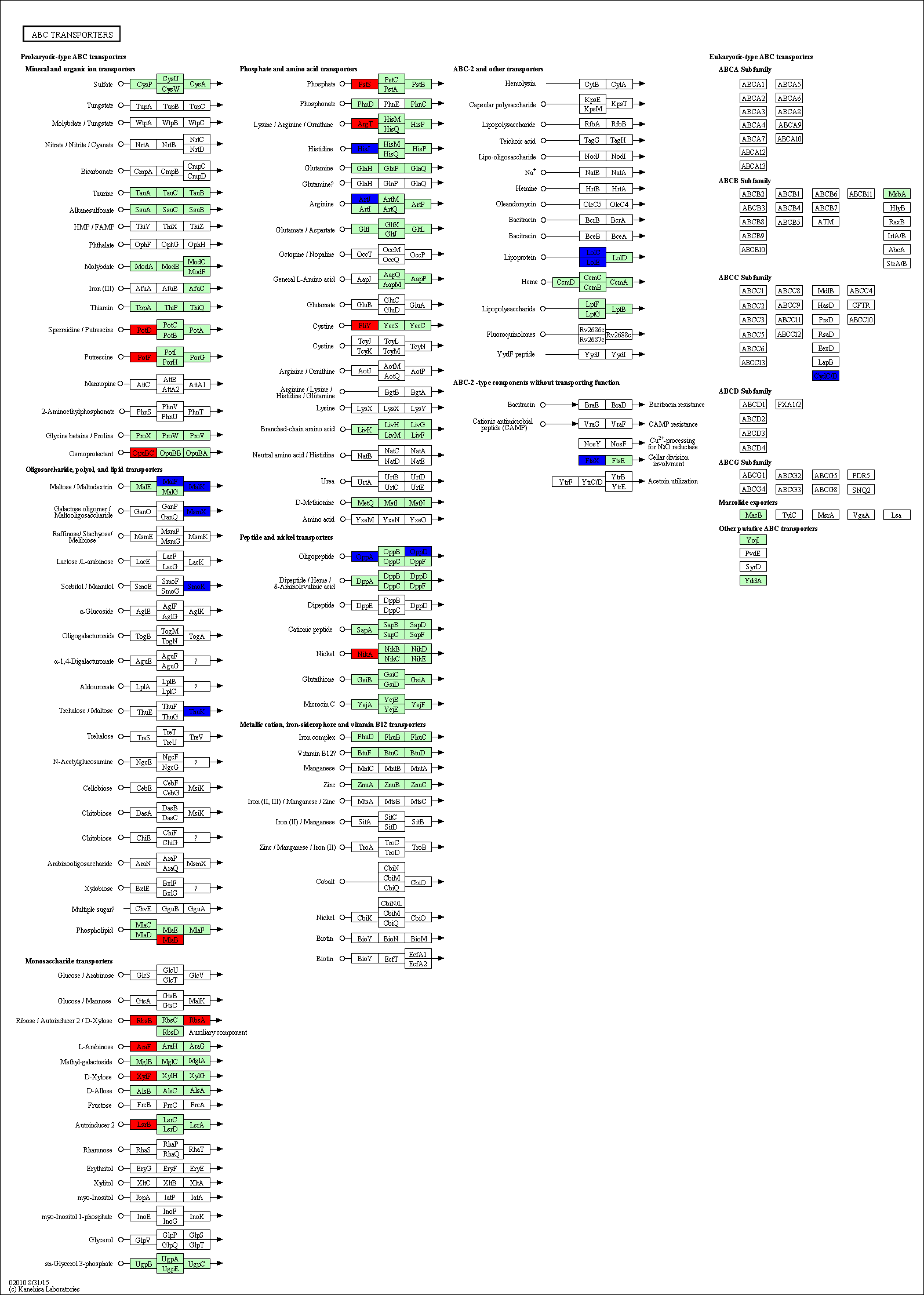
**

**S5 Fig. ABC transporters (eco02010) of p21-cDNA strain under salt stress.** Blue, down-regulated enzymes; Red, up-regulated enzymes in p21b-cDNA vs pET-21b(+). The number is the EC number of gene. AraF, subunit of arabinose ABC transporter; ArgT, subunit of lysine/arginine/ornithine ABC Transporter; ArtJ, Arginine transporter subunit; CydC/D, ABC transporter, CydDC cysteine exporter (CydDC-E) family, permease/ATP-binding protein CydC/D; FliY, Extracellular solute-binding protein family 3; FtsX, Cell division protein FtsX; HisJ, Cationic amino acid ABC transporter, periplasmic binding protein; LolC/E, Lipoprotein releasing system, transmembrane protein, LolC/E family; LsrB, ABC-type sugar transport system periplasmic component-like protein; MalF, Binding-protein-dependent transport systems inner membrane component; MalK/MsnX/SmoK/ThuK, ABC transporter related; MlaB, subunit of phospholipid ABC transporter; NikA, Nickel ABC transporter, periplasmic nickel-binding protein; OppA, Oligopeptide transporter subunit; OppD, Oligopeptide transporter ATP-binding component; OpuBC, osmF, subunit of YehW/YehX/YehY/YehZ ABC transporter; PotD, Extracellular solute-binding protein family 1; PotF, Extracellular solute-binding protein family 1; PstS, Phosphate-binding protein PstS; RbsA, ABC transporter related; RbsB, D-ribose transporter subunit; XylF, D-xylose ABC transporter, periplasmic substrate-binding.
