# Supplementary material for: Proteomic Analyses Reveal the Mechanism of Dunaliella salina Ds-26-16 Gene Enhancing Salt Tolerance in Escherichia coli
Source: PLoS One. 2016 May 2;11(5):e0153640. doi: 10.1371/journal.pone.0153640 (PMC4852897; doi:10.1371/journal.pone.0153640)
Supplement: S6 Fig — (DOC) [file pone.0153640.s006.doc]

**
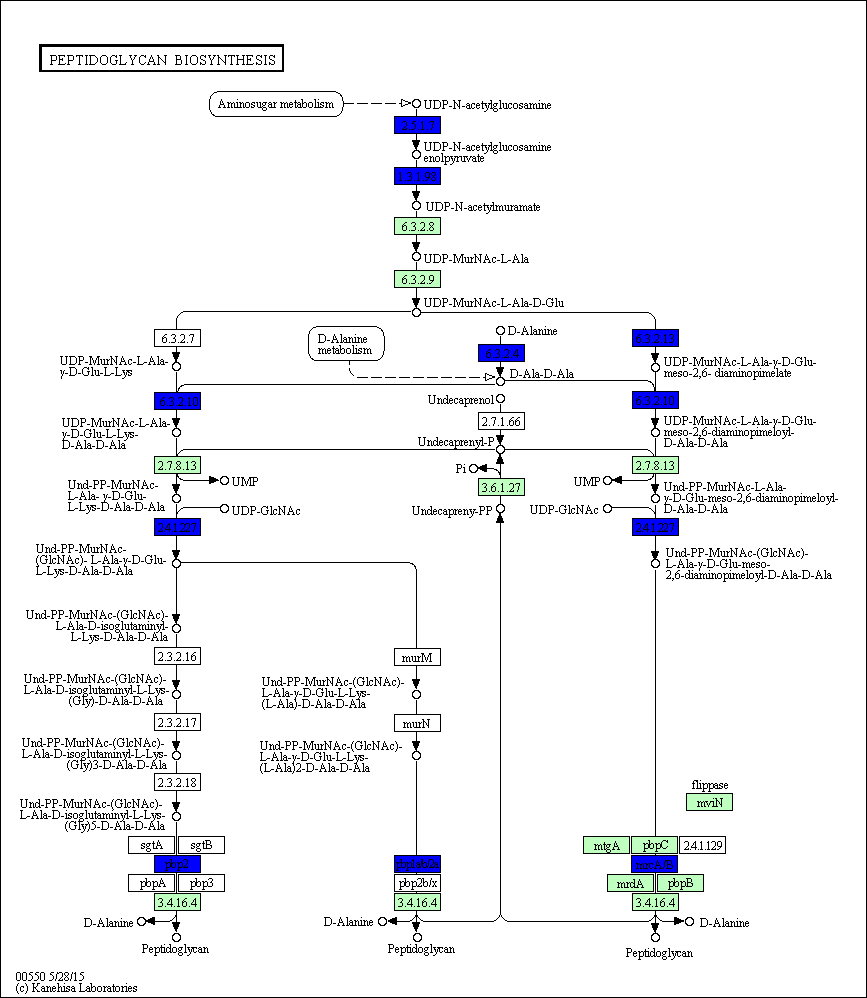
**

**S6 Fig. Peptidoglycan biosynthesis pathway (eco00550) of p21-cDNA strain under salt stress.** Blue, down-regulated enzymes; Red, up-regulated enzymes in p21b-cDNA vs pET-21b(+). The number is the EC number of gene. EC: 2.5.1.7, UDP-N-acetylglucosamine 1-carboxyvinyltransferase; EC: 1.3.1.98, UDP-N-acetylenolpyruvoylglucosamine reductase; EC: 6.3.2.13, UDP-N-acetylmuramoyl-L-alanyl-D- glutamate-2,6-diaminopimelate ligase; EC: 6.3.2.4, D-alanine-D-alanine ligase; EC: 6.3.2.10, UDP-N- acetylmuramoyl-tripeptide-D-alanyl-D-alanine ligase; EC: 2.4.1.227, UDP-N-acetylglucosamine-N- acetylmuramyl-(pentapeptide) pyrophosphoryl-undecaprenol N-acetylglucosamine transferase; mrcA/B, Fused penicillin-binding protein 1a: murein transglycosylase/murein transpeptidase; pbp2, mrcA; pbplab/2a, mrcA.
