# Supplementary material for: Proteomic Analyses Reveal the Mechanism of Dunaliella salina Ds-26-16 Gene Enhancing Salt Tolerance in Escherichia coli
Source: PLoS One. 2016 May 2;11(5):e0153640. doi: 10.1371/journal.pone.0153640 (PMC4852897; doi:10.1371/journal.pone.0153640)
Supplement: S2 Table — (PDF) [file pone.0153640.s008.pdf]

S2 Table. Differential proteins identified in p21-cDNA and p21-ORF strain under salt stress

Protein description	GO annotation	KEGG		Fold change	
		KO No.	Pathway	cDNA	ORF
**Differential proteins that showed similar expression pattern in p21-ORF and p21-cDNA**					
D-galactose transporter	CC; MF	K08137		0.137	0.124
Cytochrome b562 (Soluble)	BP; CC; MF			0.169	0.132
Ybl192	CC			0.172	0.231
Flagellar biosynthesis, hook-filament junction protein 1, subunit of Flagellum	BP; CC; MF	K02396	eco02040	0.173	0.200
Predicted metal dependent hydrolase	CC; MF	K03760		0.191	0.157
dTDP-glucose 4,6-dehydratase	BP; MF	K01710	eco00523; eco01230; eco00521; eco01110	0.193	0.173
Proline:sodium symporter	CC; MF	K11928		0.196	0.217
Ribonuclease 3	BP; CC; MF	K03685		0.202	0.187
2-dehydro-3-deoxyphosphooctonate aldolase	BP; CC; MF	K01627	eco01100; eco00540	0.203	0.160
Ybl99	BP; MF	K01790	eco00523; eco01230; eco00521; eco01110	0.205	0.234
Anti-sigma factor for FliA (Sigma 28)	BP	K02398	eco02040	0.209	0.303
Alcohol dehydrogenase GroES domain protein	MF	K00094	eco00052; eco01100	0.211	0.221
5-methyltetrahydropteroyltriglutamate-homocysteine methyltransferase	BP; MF	K00549	eco00450; eco00270; eco01230; eco00670; eco01230; eco01110	0.215	0.194
6,7-dihydropteridine reductase	MF	K10679	eco01120; eco00633	0.217	0.170
KdgR transcriptional repressor	BP; MF			0.217	0.248
Ybl13	BP; CC			0.226	0.236
Nucleotide sugar dehydrogenase	MF	K00012	eco00520; eco02060	0.227	0.234
C4-dicarboxylate transport protein	BP; CC; MF	K11103	eco02020	0.228	0.235
Translation initiation factor IF-1	CC; MF	K02518		0.228	0.254
Bifunctional polymyxin resistance protein ArnA	BP; MF	K10011	eco00520; eco01110; eco02020	0.231	0.169
Periplasmic binding protein/LacI transcriptional regulator				0.234	0.223
Glucosyltransferase I	BP; MF	K02844	eco01100; eco00540	0.239	0.309
Fused trehalose(Maltose)-specific PTS enzyme: IIB component/IIC component	BP; CC; MF	K02819	eco00500; eco01230; eco01110	0.250	0.266
GMP reductase	BP; MF	K00364	eco00230	0.251	0.278
Periplasmic binding protein/LacI transcriptional regulator	BP	K02058		0.252	0.247
Ketol-acid reductoisomerase	BP; MF	K00053	eco00290; eco00770; eco01230; eco01230; eco01110; eco01210	0.255	0.280
Acetylglutamate kinase	BP; CC; MF	K00930	eco00330; eco01230; eco01230; eco01110; eco01210	0.256	0.292
OmpA/MotB domain protein	CC			0.261	0.257
Copper/silver efflux system, outer membrane component	CC; MF	K07796	eco02020	0.266	0.317
Cytochrome bo terminal oxidase subunit II, subunit of cytochrome bo terminal oxidase	BP; CC; MF	K02297	eco00190; eco01100	0.269	0.201

Orotate phosphoribosyltransferase	BP; MF	K00762	eco01100; eco00240	0.269	0.278
Acetylornithine/succinyldiaminopimelate aminotransferase	BP; CC; MF	K00821	eco00330; eco01230; eco00480; eco01110	0.270	0.290
S-ribosylhomocysteine lyase	BP; MF	K07173	eco00270; eco01230	0.271	0.296
RNA-directed DNA polymerase	BP; MF			0.276	0.333
Arginine transporter subunit	CC; MF	K09996	eco02010	0.277	0.261
Copper/silver efflux system, membrane fusion protein	BP; CC	K07798	eco02020	0.277	0.315
Flagellar L-ring protein	BP; CC; MF	K02393	eco02040	0.277	0.567
Lipopolysaccharide core heptose(II)-phosphate phosphatase	BP; CC; MF			0.284	0.262
Argininosuccinate lyase	BP; CC; MF	K01755	eco00330; eco01230; eco00250; eco01230; eco01110	0.286	0.308
Galactitol-specific enzyme IIC component of PTS	BP; CC; MF	K02775	eco00052; eco01100; eco02060	0.289	0.226
Lysine decarboxylase	BP; CC; MF	K01582	eco00310; eco01230; eco01110	0.289	0.315
L-rhamnose isomerase	BP; CC; MF	K01813	eco00051	0.289	0.379
Carbon starvation protein CstA	BP; CC			0.290	0.340
Conserved protein				0.293	0.393
Outer membrane channel protein	BP; CC; MF	K12340	eco03070	0.294	0.378
Predicted serine transporter	CC; MF	K03837		0.296	0.437
Conserved protein		K06966		0.297	0.310
Carbamoyl-phosphate synthase small chain	BP; MF	K01956	eco00250; eco01100; eco00240	0.297	0.315
P22 repressor protein c2	MF			0.298	0.288
UDP-4-amino-4-deoxy-L-arabinose-oxoglutarate aminotransferase	BP; MF	K07806	eco00520; eco01230; eco01110	0.299	0.324
Cold-shock DNA-binding domain protein	BP; CC; MF	K03704		0.302	0.302
Carbamoyl-phosphate synthase large chain	BP; MF	K01955	eco00250; eco01100; eco00240	0.304	0.289
HTH-type transcriptional repressor PurR	BP; CC; MF	K03604		0.304	0.302
Putative uncharacterized protein ydcH		K09794		0.305	0.466
β-galactosidase	BP; CC; MF	K01190	eco00511; eco00052; eco00600; eco01230	0.306	0.274
Conserved inner membrane protein		K08996		0.308	0.418
Anaerobic ribonucleoside triphosphate reductase	BP; MF	K00527	eco00230; eco01100; eco00240	0.310	0.249
3’,5’-cyclic adenosine monophosphate phosphodiesterase CpdA	MF	K03651		0.311	0.340
Transcriptional regulator, LacI family	BP; CC; MF	K03485		0.313	0.312
(D)-galactarate dehydrogenase	BP; MF	K01708	eco00053	0.313	0.363
2-hydroxy-3-oxopropionate reductase	BP; MF	K00042	eco01100; eco00630	0.315	0.333
FepA, outer membrane receptor for ferric enterobactin (Enterochelin) and colicins B and D, subunit of Outer Membrane Ferric Enterobactin Transport System and Ferric Enterobactin Transport System	BP; CC; MF	K16089		0.315	0.340
DegT/DnrJ/EryC1/StrS aminotransferase	BP; MF			0.322	0.286
PfkB domain protein	BP; MF			0.323	0.378
Bifunctional dihydroneopterin aldolase/dihydroneopterin triphosphate 2’-epimerase	BP; MF	K01633	eco01100; eco00790	0.325	0.351
Ybl103				0.326	0.258
N-succinylglutamate 5-semialdehyde dehydrogenase	BP; MF	K06447	eco00330	0.327	0.335

Undecaprenyl-phosphate 4-deoxy-4-formamido-L-arabinose transferase	BP; CC; MF	K10012	eco00520; eco01110	0.327	0.375
Ornithine carbamoyltransferase	BP; CC; MF	K00611	eco00330; eco01230; eco01230; eco01110	0.330	0.335
Ferredoxin-NADP reductase	MF	K00528		0.330	0.407
NADPH nitroreductase monomer, subunit of NADPH nitroreductase	MF	K10678	eco01120; eco00633	0.335	0.349
Conserved inner membrane protein	CC; MF			0.335	0.375
Aspartate carbamoyltransferase	BP; MF	K00609	eco00250; eco01100; eco00240	0.338	0.415
Phosphoenolpyruvate synthase	BP; MF	K01007	eco00680; eco01200; eco01120; eco01230; eco00630	0.343	0.284
Dihydroxy-acid dehydratase	BP; MF	K01687	eco00290; eco00770; eco01230; eco01230; eco01110; eco01210	0.344	0.381
Aspartokinase	BP; MF	K00928	eco00260; eco01120; eco01230; eco01210; eco00270; eco00300; eco01100; eco01110	0.346	0.431
Galactitol-specific enzyme IIB component of PTS	BP; MF	K02774	eco00052; eco01100; eco02060	0.349	0.534
Cytochrome bo terminal oxidase subunit I, subunit of cytochrome bo terminal oxidase	BP; CC; MF	K02298	eco00190; eco01100	0.350	0.428
Major facilitator superfamily MFS-1	BP; CC			0.350	0.432
Preprotein translocase, SecG subunit	BP; CC; MF	K03075	eco03070; eco03060	0.350	0.435
DNA-binding transcriptional repressor	BP; CC; MF	K02529		0.350	0.559
dTDP-glucose 4,6-dehydratase	BP; MF	K01710	eco00523; eco01230; eco00521; eco01110	0.352	0.300
Cytoplasmic glycerophosphodiester phosphodiesterase	BP; MF	K01126	eco00564	0.358	0.429
Glucitol/sorbitol-specific enzyme IIA component of PTS	BP; CC; MF	K02781	eco00051; eco02060	0.358	0.439
B12-dependent methionine synthase	BP; CC; MF	K00548	eco00450; eco00730; eco01230	0.362	0.511
L-fucose isomerase	BP; CC; MF	K01818	eco00051	0.366	0.465
tRNA sulfurtransferase	BP; CC; MF	K03151	eco00730; eco04122; eco01230	0.371	0.450
Cobalamin synthesis protein P47K				0.372	0.389
N-acetyl-gamma-glutamyl-phosphate reductase	BP; CC; MF	K00145	eco00330; eco01230; eco01230; eco01110; eco01210	0.375	0.306
Fused penicillin-binding protein 1a: murein transglycosylase/murein transpeptidase	BP; CC; MF	K05366	eco00550; eco01230	0.375	0.400
DNA-binding protein fis	BP; MF	K03557		0.376	0.388
OmpF, subunit of outer membrane porin F and The Colicin A Import System	BP; CC; MF	K09476	eco02020	0.378	0.365
D-tagatose-1,6-bisphosphate aldolase subunit GatZ	BP; MF	K16371	eco00052; eco01100	0.382	0.361
NADH:ubiquinone oxidoreductase II	MF	K03885	eco00190	0.382	0.493
5-keto-4-deoxy-D-glucarate aldolase	BP; MF	K01630	eco00053	0.385	0.386
Hydrogenase expression/formation protein	BP; MF	K03605		0.387	0.426
N-acetylneuraminate lyase	BP; CC; MF	K01639	eco00520	0.387	0.511
Amino-acid acetyltransferase	BP; CC; MF	K14682	eco00330; eco01230; eco01230; eco01110; eco01210	0.389	0.386
Cationic amino acid ABC transporter, periplasmic binding protein	CC; MF	K10014	eco02010	0.390	0.411
Uridine kinase	BP; CC; MF	K00876	eco01100; eco00240	0.395	0.340
A,α-phosphotrehalase	BP; CC; MF	K01226	eco00500	0.397	0.315
A-galactosidase monomer, subunit of α-galactosidase	BP; MF	K07406	eco00052; eco00600; eco00561	0.398	0.431

Pyridoxamine kinase	BP; MF	K00868	eco01100; eco00750	0.400	0.427
S-adenosylmethionine synthase	BP; CC; MF	K00789	eco00270; eco01230; eco01100; eco01110	0.400	0.439
L-lactate dehydrogenase [cytochrome]	BP; MF	K00101	eco00620; eco01120; eco00627	0.400	0.461
Predicted lipoprotein		K07286		0.400	0.534
2’,3’-cyclic-nucleotide 2’-phosphodiesterase	BP; MF	K01119	eco00230; eco00240	0.407	0.422
Protein translocase subunit SecD	BP; CC; MF	K03072	eco03070; eco03060	0.411	0.505
Signal peptidase I	BP; CC; MF	K03100	eco03060	0.411	0.523
LPS-assembly protein LptD	BP; CC	K04744		0.415	0.439
3-oxoacyl-[acyl-carrier-protein] synthase 2	BP; MF	K09458	eco00780; eco00061; eco01230; eco01212	0.416	0.504
Conserved protein		K14762		0.416	0.577
Nucleoside (Except guanosine) transporter	CC; MF	K11535		0.418	0.473
S-adenosylmethionine:tRNA ribosyltransferase-isomerase	BP; CC; MF	K07568		0.419	0.316
Predicted oxidoreductase				0.420	0.359
UPF0265 protein YeeX		K09802		0.420	0.571
Lipopolysaccharide export system protein LptC	BP; CC; MF	K11719		0.422	0.561
Transketolase	MF	K00615	eco01200; eco01120; eco01230; eco01230; eco01110; eco00030	0.423	0.491
Cof-like hydrolase	MF	K07024		0.426	0.371
NADH-quinone oxidoreductase subunit A	BP; CC; MF	K00330	eco00190; eco01100	0.426	0.487
Aspartate-ammonia ligase	BP; CC; MF	K01914	eco01230; eco00250; eco01230; eco01110; eco00460	0.428	0.474
NADH-quinone oxidoreductase subunit B	BP; CC; MF	K00331	eco00190; eco01100	0.429	0.466
UDP-N-acetylenolpyruvoylglucosamine reductase	BP; CC; MF	K00075	eco00520; eco00550; eco01230	0.431	0.315
EnvC murein hydrolase	BP; MF			0.435	0.472
D-alanyl-D-alanine carboxypeptidase	BP; MF	K07259		0.436	0.455
Oligopeptide transporter ATP-binding component	BP; CC; MF	K15583	eco02010	0.438	0.449
Phosphorylase	BP; MF	K00688	eco00500; eco01230; eco01110	0.438	0.505
Aspartate-semialdehyde dehydrogenase	BP; CC; MF			0.439	0.312
Cell division protein DamX	BP	K03112		0.441	0.574
Delta-1-pyrroline-5-carboxylate dehydrogenase	BP; MF	K13821	eco00330; eco00250; eco01230; eco01110	0.442	0.417
Cell divisionFtsK/SpoIIIE	BP; CC; MF			0.446	0.490
Ribosomal silencing factor RsfS		K09710		0.449	0.422
3-dehydroquinate synthase	BP; CC; MF	K01735	eco00400; eco01230; eco01230; eco01110	0.449	0.427
Aminotransferase class I and II	BP; MF	K00835	eco00290; eco01230; eco01110	0.450	0.405
ABC transporter, CydDC cysteine exporter (CydDC-E) family, permease/ATP-binding protein CydD	BP; CC; MF	K16013	eco02010	0.451	0.546
NADH-quinone oxidoreductase, F subunit	MF	K00335	eco00190; eco01100	0.453	0.516
Membrane protein insertase YidC	BP; CC	K03217	eco03070; eco03060	0.454	0.578
DNA-binding transcriptional dual regulator	BP; CC; MF	K03435		0.455	0.448
Cys-tRNA(Pro)/Cys-tRNA(Cys) deacylase				0.457	0.351

Delta-aminolevulinic acid dehydratase	BP; MF	K01698	eco00860; eco01230; eco01110	0.458	0.507
Outer membrane protein X	CC	K11934		0.460	0.622
Cytochrome-c3 hydrogenase	MF	K06281	eco01120; eco00633	0.461	0.529
ProP effector	CC	K03607		0.463	0.344
ABC transporter, CydDC cysteine exporter (CydDC-E) family, permease/ATP-binding protein CydC	BP; CC; MF	K16012	eco02010	0.466	0.504
Conserved protein, subunit of EfeU/EfeO/EfeB ferrous iron transporter cryptic		K07224		0.466	0.562
Fructose-6-phosphate aldolase	BP; CC; MF	K08314		0.466	0.582
Maltoporin	BP; CC; MF	K02024		0.467	0.474
Protein-export membrane protein SecF	BP; CC; MF	K03074	eco03070; eco03060	0.467	0.521
GTP cyclohydrolase 1	BP; CC; MF	K01495	eco01100; eco00790	0.467	0.574
Pal, subunit of The Tol-Pal Cell Envelope Complex, Colicin S4 Transport System and The Colicin A Import System	CC	K03640		0.467	0.575
Pseudouridine synthase	BP; MF	K06179		0.469	0.563
Cytochrome bd ubiquinol oxidase subunit I	CC; MF	K00425	eco00190; eco01100; eco02020	0.470	0.535
DNA binding protein, nucleoid-associated	BP; CC; MF	K11685		0.471	0.477
Conserved protein involved in bacteriophage adsorption				0.471	0.583
Branched-chain amino acid aminotransferase	BP; MF	K00826	eco00280; eco00290; eco00770; eco01230; eco01100; eco01110; eco01210	0.472	0.478
Lipoprotein releasing system, transmembrane protein, LolC/E family	CC; MF	K09808	eco02010	0.474	0.561
ABC transporter related	BP; CC; MF	K10111	eco02010	0.475	0.447
Outer membrane phospholipase A	BP; CC; MF	K01058	eco00565; eco00590; eco01230; eco00564; eco00592	0.475	0.544
Glutamate-1-semialdehyde 2,1-aminomutase	BP; CC; MF	K01845	eco00860; eco01230; eco01110	0.476	0.492
NAD(P)H dehydrogenase (Quinone)	MF	K03923		0.478	0.482
Binding-protein-dependent transport systems inner membrane component	CC; MF	K10109	eco02010	0.480	0.515
NADH-quinone oxidoreductase subunit N	BP; CC; MF	K00343	eco00190; eco01100	0.480	0.602
Probable phosphoglycerate mutase GpmB	BP; MF	K15634	eco00680; eco00260; eco01120; eco01230; eco01200; eco01230; eco00010; eco01110	0.481	0.447
Glycerate kinase I	BP; MF	K00865	eco00260; eco01100; eco01110; eco00561; eco00630	0.484	0.517
Aspartate ammonia-lyase	BP; MF	K01744	eco00250; eco01100	0.485	0.442
NADH-quinone oxidoreductase, chain G	BP; CC; MF	K00336	eco00190; eco01100	0.485	0.570
Conserved protein				0.486	0.501
UDP-N-acetylmuramoyl-tripeptide-D-alanyl-D-alanine ligase	BP; CC; MF	K01929	eco00300; eco00550; eco01230	0.487	0.326
Conserved protein		K09136		0.489	0.352
Lipoyl synthase	BP; CC; MF	K03644	eco00785; eco01230	0.490	0.487
UDP-N-acetylmuramoyl-L-alanyl-D-glutamate-2,6-diaminopimelate ligase	BP; CC; MF	K01928	eco00300; eco00550	0.494	0.520
Na^+/H^+ antiporter	CC; MF	K03316		0.496	0.476
Protein YcgL		K09902		0.496	0.613

NADH-quinone oxidoreductase subunit C/D	BP; CC; MF	K13378	eco00190; eco01100	0.499	0.528
ATP-dependent dsDNA exonuclease, subunit of SbcCD ATP-dependent dsDNA exonuclease	BP; MF	K03546		0.502	0.510
Maltose O-acetyltransferase	MF	K00661		0.506	0.568
3-phosphoserine phosphatase	BP; MF	K01079	eco00680; eco01200; eco00620; eco01120; eco00010; eco01230; eco01110; eco00640	0.508	0.434
Membrane spanning protein in TolA-TolQ-TolR complex	CC; MF	K03562	eco01120	0.508	0.499
ATP synthase subunit b	BP; CC; MF	K02109	eco00190; eco01100	0.510	0.523
Aromatic-amino-acid transaminase	BP; MF	K00832	eco00350; eco00360; eco00270; eco00400; eco01230; eco01230; eco01110; eco00401	0.514	0.525
Cysteine-tRNA ligase	BP; CC; MF	K01883	eco00970	0.514	0.566
Efflux pump membrane protein	CC; MF	K03543		0.517	0.547
2-oxoglutarate dehydrogenase, E1 subunit	BP; MF	K00164	eco01200; eco01120; eco00310; eco00020; eco01230; eco01110; eco00380	0.518	0.420
Fumarase A monomer, subunit of fumarase A	BP; MF	K01676	eco01200; eco01120; eco00020; eco01230; eco01110	0.518	0.462
7,8-dihydropteroate synthase	BP; MF	K00796	eco01100; eco00790	0.518	0.523
AcrA membrane fusion protein, subunit of AcrAB-TolC multidrug efflux transport system and AcrAD-TolC multidrug efflux transport system	BP; CC	K03585		0.518	0.592
LPS-assembly lipoprotein LptE	BP; CC	K03643		0.521	0.622
UDP-N-acetylglucosamine-N-acetylmuramyl-(pentapeptide) pyrophosphoryl-undecaprenol N-acetylglucosamine transferase	BP; CC; MF	K02563	eco00550; eco01230	0.522	0.530
Oligopeptide transporter subunit	MF	K15580	eco02010	0.523	0.508
Sec-independent protein translocase protein TatA	BP; CC; MF	K03116	eco03070; eco03060	0.523	0.538
tRNA pseudouridine synthase A	BP; MF	K06173		0.526	0.194
Negative modulator of initiation of replication	BP; CC; MF	K03645		0.526	0.543
MazG family protein	MF	K04765	eco00230; eco01100; eco00240	0.526	0.551
D-serine dehydratase	BP; MF	K01753	eco00260	0.529	0.603
MlaA	CC	K04754		0.532	0.621
L-threonine 3-dehydrogenase	BP; CC; MF	K00060	eco00260	0.534	0.592
2,3,4,5-tetrahydropyridine-2,6-dicarboxylate N-succinyltransferase	BP; CC; MF	K00674	eco01120; eco00300; eco01230; eco01230	0.535	0.613
Predicted rhodanese-related sulfurtransferase	MF			0.539	0.571
Aspartate kinase	BP; MF	K12524	eco00270; eco00260; eco01120; eco00300; eco01230; eco01100; eco01110	0.540	0.497
NADH-quinone oxidoreductase subunit I	BP; CC; MF	K00338	eco00190; eco01100	0.540	0.566
Putative sulfatase	MF	K01138		0.540	0.600
Cell division protein FtsX	BP; CC	K09811	eco02010	0.540	0.622
Cytochrome bd-I terminal oxidase subunit II, subunit of cytochrome bd-I terminal oxidase	CC; MF	K00426	eco00190; eco01100; eco02020	0.543	0.544

Aspartate kinase	BP; MF	K12525	eco00270; eco00260; eco01120; eco00300; eco01230; eco01100; eco01110	0.544	0.593
Succinate dehydrogenase and fumarate reductase iron-sulfur protein	BP; MF	K00240	eco01120; eco00623; eco00020; eco01200; eco00190; eco00650; eco01230; eco01110	0.544	0.598
Orotidine 5’-phosphate decarboxylase	BP; MF	K01591	eco01100; eco00240	0.544	0.629
β-lactamase	BP; MF	K07258		0.545	0.578
DNA-binding transcriptional repressor	MF	K15545		0.549	0.591
Dual-specificity RNA methyltransferase RlmN	BP; CC; MF	K06941		0.552	0.544
Aspartate carbamoyltransferase regulatory chain	BP; CC; MF	K00610	eco00250; eco01100; eco00240	0.553	0.554
Succinate dehydrogenase cytochrome b556 small membrane subunit	BP; CC; MF	K00242	eco01120; eco00623; eco00020; eco01200; eco00190; eco00650; eco01230; eco01110	0.555	0.553
Predicted DNA-binding transcriptional regulator	MF			0.556	0.571
4-α-glucanotransferase (Amylomaltase)	MF	K00705	eco00500; eco01230	0.560	0.464
Ribosome maturation factor RimM	BP; CC; MF	K02860		0.560	0.582
HU, DNA-binding transcriptional regulator, β subunit	BP; MF	K03530		0.560	0.609
Cof-like hydrolase	MF	K07024		0.561	0.447
Dihydrolipoamide acetyltransferase	BP; CC; MF	K00627	eco01200; eco00620; eco01120; eco00020; eco00010; eco01230; eco01110	0.561	0.600
6-phosphogluconate dehydratase	BP; MF	K01690	eco01200; eco01120; eco01230; eco00030	0.562	0.481
2-amino-3-ketobutyrate CoA ligase	BP; MF	K00639	eco00260	0.564	0.600
Carbonic anhydrase	BP; MF	K01673	eco00910	0.564	0.655
Outer membrane protein assembly factor BamA	BP; CC	K07277		0.565	0.547
Thioredoxin reductase	BP; CC; MF	K00384	eco00450; eco00240	0.565	0.654
Ribonuclease T	BP; CC; MF	K03683		0.568	0.497
UDP-N-acetylglucosamine 1-carboxyvinyltransferase	BP; CC; MF	K00790	eco00520; eco00250; eco01230; eco01110	0.568	0.511
Predicted NAD(P)-binding dehydrogenase	BP; MF			0.570	0.427
Uridylate kinase	BP; CC; MF	K09903	eco01100; eco00240	0.570	0.628
Peptidase domain protein				0.572	0.640
PabB, subunit of aminodeoxychorismate synthase and para-aminobenzoate synthase multi-enzyme complex	BP; MF	K01665	eco00790	0.575	0.652
Outer membrane protein assembly factor BamD	BP; CC	K05807		0.575	0.654
AgaR transcriptional repressor	BP; CC; MF	K02081		0.575	0.667
tRNA pseudouridine synthase D	BP; MF	K06176		0.576	0.590
Outer membrane protein assembly factor BamB	BP; CC	K17713		0.579	0.556
Cell wall structural complex MreBCD transmembrane component MreC	BP; CC	K03570		0.579	0.628
2-oxoglutarate dehydrogenase, E2 subunit, dihydrolipoamide succinyltransferase	BP; CC; MF	K00658	eco01200; eco01120; eco00310; eco00020; eco01230; eco01110	0.579	0.621
Riboflavin synthase	BP; MF	K00793	eco00740; eco01230	0.581	0.415
Mammalian cell entry related domain protein		K06192		0.581	0.648
Outer-membrane lipoprotein LolB	CC; MF	K02494		0.583	0.664

tRNA pseudouridine synthase B	BP; MF	K03177		0.584	0.503
Peptidyl-prolyl cis-trans isomerase	BP; MF	K03773		0.585	0.651
Fimbrial biogenesis outer membrane usher protein	CC; MF	K07347		0.588	0.486
Extracellular solute-binding protein family 3	CC; MF	K10001	eco02010; eco02020	0.589	0.569
Acetylornithine deacetylase	BP; CC; MF	K01438	eco00330; eco01230; eco01230; eco01110; eco01210	0.589	0.665
DNA-binding transcriptional repressor	BP; CC; MF	K02529		0.593	0.456
Glutamine-fructose-6-phosphate aminotransferase [isomerizing]	BP; CC; MF	K00820	eco00520; eco00500; eco01230; eco01110	0.596	0.487
Bifunctional protein FolC	BP; MF	K11754	eco01100; eco00790	0.599	0.540
Ornithine decarboxylase, biosynthetic	BP; CC; MF	K01581	eco00330; eco01120; eco00300; eco01230; eco01230; eco01110; eco01210	0.601	0.526
D-alanine-D-alanine ligase	BP; CC; MF	K01921	eco00473; eco00550; eco01230	0.604	0.619
Hydrolase, TatD family	MF	K03424		0.605	0.565
Oxidoreductase domain protein	MF			0.612	0.537
NADH-quinone oxidoreductase subunit K	BP; CC; MF	K00340	eco00190; eco01100	0.615	0.656
Arabinose 5-phosphate isomerase	BP; MF	K02467		0.623	0.575
ATP-dependent RNA helicase RhlB	BP; MF	K03732	eco03018	0.632	0.522
Formamidopyrimidine-DNA glycosylase	BP; MF	K10563	eco03410	0.634	0.635
Elongation factor 4	BP; CC; MF	K03596		0.635	0.585
AcrB RND-type permease, subunit of AcrAB-TolC multidrug efflux transport system	CC; MF	K03296		0.640	0.532
N-ethylmaleimide reductase, FMN-linked	MF	K10680	eco01120; eco00633	0.640	0.594
Chaperone, periplasmic	CC	K07346		0.644	0.510
Peptide chain release factor 1	CC; MF	K02835		0.645	0.603
Pseudouridine synthase	BP; MF	K06183		0.650	0.628
Pyruvate dehydrogenase E1 component	MF	K00163	eco01200; eco00620; eco01120; eco00020; eco00010; eco01230; eco01110	0.651	0.531
Alanine racemase	BP; MF	K01775	eco00473; eco01230	0.651	0.572
HAD-superfamily hydrolase, subfamily IA, variant 3	MF	K01112	eco00051	0.653	0.391
Conserved protein				0.654	0.660
tRNA uridine 5-carboxymethylaminomethyl modification enzyme MnmG	BP; CC; MF	K03495		0.656	0.440
Putative Holliday junction resolvase	BP; CC; MF	K07447		0.656	0.653
Methylated adenine and cytosine restriction protein	BP; MF	K07448		0.657	0.364
Hypoxanthine-guanine phosphoribosyltransferase	BP; CC; MF	K00760	eco00230; eco01100; eco01110	0.664	0.584
Ubiquinone biosynthesis O-methyltransferase	BP; MF	K00568	eco01100; eco01212; eco00071	0.670	0.529
LYSR-type transcriptional regulator	BP; MF			1.474	1.547
Kch potassium VIC channel subunit, subunit of Kch potassium VIC channel	BP; CC; MF	K10716		1.497	1.752
Putative uncharacterized protein yebV				1.498	1.862
Glyoxalase I	MF	K01759	eco00620	1.500	1.613
Phosphate-binding protein PstS	CC; MF	K02040	eco02010; eco02020	1.524	1.886
ATP-dependent chaperone ClpB	BP; CC; MF	K03695		1.535	1.494

Xylulokinase	BP; MF	K00854	eco01100; eco00040	1.537	1.605
Multicopper oxidase (Laccase)	MF	K14588		1.542	1.678
Putative uncharacterized protein yggN				1.547	1.581
Dihydroxyacetone kinase subunit K, subunit of dihydroxyacetone kinase	BP; MF	K05878	eco01100; eco00561	1.550	1.523
ArgT, subunit of lysine/arginine/ornithine ABC Transporter	CC; MF	K10013	eco02010	1.552	1.464
UPF0307 protein YjgA		K09889		1.553	1.817
Maf-like protein YceF	CC			1.557	1.892
Superoxide dismutase	BP; MF	K04564		1.563	1.655
Peptidase M16 domain protein	BP; MF	K01407		1.564	1.719
N-succinylarginine dihydrolase	BP; MF	K01484	eco00330	1.592	1.544
2-keto-3-deoxygluconokinase	MF	K00874	eco01200; eco01120; eco01230; eco00030	1.600	1.697
Predicted ribosome-associated, sigma 54 modulation protein	BP; CC	K05808		1.602	1.740
MoaB subunit, subunit of molybdopterin biosynthesis protein B	BP	K03638		1.614	1.811
Aldo/keto reductase	MF			1.618	1.764
Tryptophanase	MF	K01667	eco00380	1.632	1.591
Extracellular solute-binding protein family 1	CC; MF	K11069	eco02010	1.638	1.791
Pirin-like protein	MF	K06911		1.656	1.678
Conserved protein				1.661	7.161
1,4-α-glucan branching enzyme GlgB	BP; MF	K00700	eco00500; eco01230; eco00040	1.665	1.816
Phosphopentomutase	BP; CC; MF	K01839	eco00230; eco00030	1.666	1.528
Fumarate reductase (Anaerobic), Fe-S subunit	BP; MF	K00245	eco01120; eco00623; eco00020; eco02020; eco01200; eco00190; eco00650; eco01230; eco01110	1.670	1.880
Glucose-1-phosphate adenylyltransferase	BP; MF	K00975	eco00520; eco00500; eco01230; eco01110; eco00053; eco00040	1.681	1.724
Filamentation induced by cAMP protein Fic	BP	K04095		1.701	1.939
Disulfide isomerase/thiol-disulfide oxidase	BP; MF	K03805		1.729	2.081
Extracellular solute-binding protein family 1	MF	K05813	eco02010	1.731	1.765
Glutathione transferase	MF	K07393		1.744	1.903
Cytidine deaminase	MF	K01489	eco01100; eco00240	1.744	1.917
Ribose-phosphate pyrophosphokinase	BP; CC; MF	K00948	eco01200; eco01120; eco00230; eco01230; eco01230; eco01110; eco00030	1.758	1.567
Asparaginase II	BP; MF	K01424	eco00250; eco01100; eco01110; eco00460	1.766	1.577
Probable endonuclease 4	BP; CC; MF	K01151	eco03410	1.767	1.677
Predicted oxidoreductase				1.769	1.926
AraF, subunit of arabinose ABC transporter	BP; MF	K10537	eco02010	1.782	1.609
Conserved protein				1.784	1.757
UPF0270 protein YheU		K09898		1.797	2.584
Nitrate reductase 1, β (Fe-S) subunit	BP; CC; MF	K00371	eco01120; eco00910; eco02020	1.808	2.195
Catalase-peroxidase	BP; MF	K03782	eco00360; eco01120; eco01230	1.816	1.542

Glutathione S-transferase	MF	K00799	eco00480	1.855	1.838
Acyl-CoA thioesterase II	BP; MF	K10805	eco01040	1.862	1.918
5'-Nucleotidase domain protein	BP; MF	K11751	eco00230; eco00760; eco01100; eco00240; eco01110	1.865	1.930
MlaB, subunit of phospholipid ABC transporter		K07122		1.867	3.130
ChbB, subunit of EIIChb	BP; MF	K02760	eco02060	1.884	2.088
Glycerol kinase	BP; MF	K00864	eco01100; eco00561	1.888	1.990
Anaerobic dimethyl sulfoxide reductase, A subunit, DmsA/YnfE family	MF	K07306	eco00920	1.890	2.230
DNA-binding transcriptional activator	BP; MF	K03717		1.895	2.046
Glutathione S-transferase domain protein	MF			1.902	1.689
Predicted DNase	MF	K03424		1.902	1.892
Putative uncharacterized protein yggL		K09923		1.915	2.453
Predicted transcriptional regulator	BP; MF			1.927	2.008
Thymidine phosphorylase	BP; MF	K00758	eco01100; eco00240	1.931	1.809
Galactoside O-acetyltransferase	MF	K00633		1.936	1.866
Esterase FrsA	MF	K11750		1.943	1.886
Carboxylate-amine ligase YbdK	BP; MF	K06048		1.945	2.023
Phosphofructokinase	MF	K00882	eco00051	1.946	1.519
Conserved protein				1.969	2.263
Conserved inner membrane protein				1.988	1.945
Sigma factor-binding protein Crl	BP; CC; MF			2.028	1.492
Predicted DNA-binding transcriptional regulator	BP; MF			2.046	1.902
Glutathione S-transferase domain protein	MF			2.052	2.036
Stress-induced protein	BP	K14055		2.078	1.653
Altronate oxidoreductase	MF	K00041	eco01100; eco00040	2.084	2.036
Murein peptide amidase A	BP; MF	K14054		2.095	2.145
N5-carboxyaminoimidazole ribonucleotide mutase	BP; MF	K01588	eco00230; eco01100; eco01110	2.123	2.629
A-ketoglutarate transporter	CC; MF	K03761		2.163	2.536
2-dehydro-3-deoxyphosphogluconate aldolase/4-hydroxy-2-oxoglutarate aldolase	MF	K01625	eco01200; eco00330; eco01120; eco01230; eco00030; eco00630	2.187	2.105
Alkylphosphonate utilization operon protein PhnA		K06193	eco01120; eco00440	2.188	2.449
Hydrogenase (NiFe) small subunit HydA	CC; MF	K06282	eco01120; eco00633	2.197	2.240
D-malate dehydrogenase (Decarboxylating)	MF	K07246	eco00650; eco00410; eco00250; eco01230; eco01110; eco00430	2.204	2.734
3-isopropylmalate dehydratase small subunit	BP; CC; MF	K01704	eco00290; eco00660; eco01230; eco01230; eco01110; eco01210	2.209	2.214
Periplasmic trehalase	BP; CC; MF	K01194	eco00500; eco02060	2.213	2.335
ABC transporter related	BP; CC; MF	K10441	eco02010	2.221	1.899
Lipoate-protein ligase A	BP; CC; MF	K03800	eco00785; eco01230	2.236	1.778
Exoribonuclease 2	BP; CC; MF	K01147		2.253	1.691

Periplasmic nitrate reductase	BP; CC; MF	K02567	eco01120; eco00910	2.262	2.405
Glycerol-3-phosphate dehydrogenase (Anaerobic), small subunit, subunit of glycerol-3-phosphate-dehydrogenase, anaerobic	BP; CC; MF	K00113	eco00564	2.269	2.104
Molybdenum cofactor synthesis domain protein	BP	K03750		2.270	1.742
Putative uncharacterized protein ydeI				2.272	3.090
BolA transcriptional dual regulator		K05527		2.284	2.199
Deoxyribose-phosphate aldolase	BP; CC; MF	K01619	eco00030	2.285	2.546
Nitrate reductase 2 (NRZ), α subunit	BP; CC; MF	K00370	eco01120; eco00910; eco02020	2.301	2.816
Phosphoribosylamine-glycine ligase	BP; MF	K01945	eco00230; eco01100; eco01110	2.304	1.745
MdtF, subunit of MdtEF-Tolc multidrug efflux transport system	CC; MF	K03296		2.312	2.427
Conserved protein	MF			2.315	3.353
Aldehyde Dehydrogenase	MF	K07248	eco00620; eco01120; eco01230; eco01200; eco00230; eco01230; eco00010; eco01110	2.318	2.137
HTH-type transcriptional regulator IscR	BP; MF	K13643		2.332	1.703
Conserved protein				2.336	2.247
Ribokinase	BP; MF	K00852	eco00030	2.377	2.079
ATP-dependent dethiobiotin synthetase BioD	BP; CC; MF	K01935	eco00780; eco01230	2.379	2.800
Conserved protein		K09807		2.382	2.580
Phosphoribosylglycinamide formyltransferase 2	BP; MF	K08289	eco00230; eco00670; eco01100	2.391	2.122
7-α-hydroxysteroid dehydrogenase	MF	K00076		2.417	2.570
Amidophosphoribosyltransferase	BP; MF	K00764	eco00230; eco00250; eco01100; eco01110	2.421	2.831
Predicted enzyme	MF			2.423	2.823
Putative uncharacterized protein yoaC				2.435	2.222
Acid phosphatase	MF	K01093	eco00562; eco00740; eco01120; eco00627	2.442	2.087
Acidic protein that suppresses mutants lacking function of protein export		K12147		2.444	2.705
Alcohol dehydrogenase zinc-binding domain protein	MF	K00344		2.448	2.480
DNA-binding response regulator in two-component regulatory system with KdpD	BP; MF	K07667	eco02020	2.460	2.290
Chorismate mutase	BP; CC; MF	K14170	eco00400; eco01230; eco01230; eco01110	2.467	2.040
Iron-sulfur cluster binding protein	BP; MF			2.483	2.642
Alcohol dehydrogenase zinc-binding domain protein	MF	K07119		2.494	3.176
Microcompartments protein	BP	K04026		2.502	2.150
Anaerobic class I fumarate hydratase (Fumarase B)	BP; MF	K01676	eco01200; eco01120; eco00020; eco01230; eco01110	2.527	2.534
Nickel ABC transporter, periplasmic nickel-binding protein	MF	K15584	eco02010	2.532	1.968
Periplasmic protein combats stress	CC	K06006		2.539	2.967
DNA protection during starvation protein	BP; CC; MF	K04047		2.545	2.593
L-1,2-propanediol oxidoreductase	MF	K00048	eco00620; eco01120; eco00630	2.550	2.591
Predicted thiosulfate sulfur transferase	MF	K01011	eco00920; eco02010	2.558	2.460
D-xylose ABC transporter, periplasmic substrate-binding protein	BP; MF	K10543	eco02010	2.583	1.972
Molecular chaperone Hsp31 and glyoxalase 3	BP; CC; MF	K05523		2.605	2.540

Dihydrofolate reductase/dihydromonapterin reductase	MF	K13938	eco00670; eco01230; eco00790	2.621	2.925
Isocitrate lyase	BP; MF	K01637	eco01200; eco01120; eco01230; eco00630	2.623	1.931
CoA-binding domain protein	MF	K06929		2.624	2.441
Curved DNA-binding protein	BP; CC; MF	K05516		2.630	2.140
NAD-dependent protein deacylase	MF	K12410		2.632	2.620
D-ribose transporter subunit	MF	K10439	eco02010; eco02030	2.637	2.957
Envelope stress induced periplasmic protein	CC			2.637	4.549
Extracellular solute-binding protein family 1	CC; MF	K11073	eco02010	2.654	2.482
AsmA family protein		K07290		2.689	2.589
Flagellin domain protein	BP; CC; MF	K02406	eco02040; eco02020	2.696	6.044
Cysteine desulfurase	BP; CC; MF	K11717	eco00450; eco00270; eco01230; eco01230; eco01110	2.716	2.212
Acyl-CoA dehydrogenase	BP; MF	K06445	eco01100; eco01110; eco00130	2.722	3.143
Aldo-keto reductase	MF			2.724	2.969
4-deoxy-L-threo-5-hexosulose-uronate ketol-isomerase	BP; MF	K01815	eco00040	2.730	3.020
AsnC transcriptional dual regulator	BP; CC; MF	K03718		2.740	2.818
Nitrate reductase 1, α subunit	BP; CC; MF	K00370	eco01120; eco00910; eco02020	2.744	2.712
Putative uncharacterized protein yccJ				2.754	3.076
Gamma-aminobutyraldehyde dehydrogenase	BP; MF	K00137	eco00330; eco00410	2.760	2.491
Acyl-CoA dehydrogenase domain protein	MF	K09456		2.780	3.468
Conserved protein	MF	K04750		2.796	3.171
Nitrate reductase 2 (NRZ), β subunit	BP; CC; MF	K00371	eco01120; eco00910; eco02020	2.811	3.070
DNA-binding transcriptional activator	CC; MF	K04064		2.814	3.003
Peptidoglycan-binding LysM	BP			2.833	3.418
Phosphoribosylaminoimidazole-succinocarboxamide synthase	BP; MF	K01923	eco00230; eco01100; eco01110	2.859	1.690
Bifunctional purine biosynthesis protein PurH	BP; MF	K00602	eco00230; eco00670; eco01100	2.862	2.942
Cysteine desulfurase ATPase component	BP; MF	K09013		2.887	2.026
Cold shock protein E	BP; CC; MF	K03704		2.890	3.210
Conserved protein	MF			2.909	3.807
PEBP family protein	MF	K06910		2.916	2.933
Gamma-glutamyltransferase	BP; MF	K00681	eco00590; eco01230; eco00480; eco00460; eco00430	2.954	3.067
ABC-type sugar transport system periplasmic component-like protein	BP	K10555	eco02010	2.967	2.574
Glutathione S-transferase domain protein	MF	K11209		2.969	3.085
Putative uncharacterized protein ygiW				2.975	3.162
4-aminobutyrate aminotransferase	BP; CC; MF	K07250	eco00280; eco00650; eco00410; eco00250; eco01100; eco00640	2.977	2.989
Extracellular solute-binding protein family 1	BP; CC; MF	K02055		2.979	2.948
Peptidase T	BP; CC; MF	K01258		3.018	3.030
D-cysteine desulfhydrase	BP; MF	K05396	eco00270	3.032	3.374

CinA domain protein				3.071	2.772
Lon protease	BP; CC; MF	K01338		3.074	1.982
Putative mannosyl-3-phosphoglycerate phosphatase	BP; CC; MF	K07026	eco00051	3.101	1.875
Acid-resistance protein, possible chaperone, subunit of HdeA dimer, inactive form of acid-resistance protein				3.113	4.323
Bacterioferritin	BP; MF	K03594		3.130	3.181
Succinate semialdehyde dehydrogenase, $NADP^+$-dependent	BP; CC; MF	K00135	eco00350; eco00650; eco01120; eco00310; eco00250; eco01100	3.141	2.888
Predicted ATPase		K06915		3.173	2.610
Putative structural protein	MF			3.177	3.980
Conserved protein				3.182	2.790
Glycerol-3-phosphate dehydrogenase (Anaerobic), large subunit, subunit of glycerol-3-phosphate-dehydrogenase, anaerobic	BP; CC; MF	K00111	eco00564	3.202	1.796
Trans-aconitate 2-methyltransferase	CC; MF	K00598		3.208	3.598
Sulfoxide reductase catalytic subunit YedY	CC; MF	K07147		3.215	4.022
Succinylornithine transaminase	BP; MF	K00840	eco00330	3.220	2.703
Anaerobic glycerol-3-phosphate dehydrogenase subunit B	BP; MF	K00112	eco00564	3.239	1.909
Aldo/keto reductase	MF			3.249	2.342
Inhibitor of vertebrate C-lysozyme	BP; CC			3.263	3.092
Glycerol 3-phosphate dehydrogenase, aerobic	BP; CC; MF	K00111	eco00564	3.288	3.324
Efflux transporter, RND family, MFP subunit	BP; CC			3.368	3.595
Lipopolysaccharide transport periplasmic protein LptA	BP; CC; MF	K09774		3.403	3.793
Glutaredoxin 2 (Grx2)	BP; MF	K03675		3.443	3.203
Nucleotide binding filament protein	BP; MF	K14061		3.443	3.356
HtrA suppressor protein				3.448	4.164
Nitrate reductase 1, gamma (Cytochrome b(NR)) subunit	CC; MF	K00374	eco01120; eco00910; eco02020	3.457	3.537
NAD(P)H dehydrogenase (quinone)	BP; MF	K03809		3.519	3.590
Hydrogenase 1, large subunit	MF	K06281	eco01120; eco00633	3.536	3.789
Acid stress chaperone				3.553	3.453
Acetaldehyde dehydrogenase	MF	K00138		3.557	3.401
Glutathione peroxidase	BP; MF	K00432	eco00590; eco00480	3.572	2.310
Glutaminase	BP; CC; MF	K01425	eco00330; eco00250; eco01100; eco00471	3.626	3.772
Conserved protein				3.651	5.009
Osmotically inducible peroxidase OsmC	BP; MF	K04063		3.660	4.239
Phosphoadenosine phosphosulfate reductase	BP; CC; MF	K00390	eco00920; eco00270; eco01120; eco04122; eco01230	3.667	1.741
Fructose bisphosphate aldolase monomer, subunit of fructose bisphosphate aldolase class I	BP; MF	K11645	eco00680; eco01200; eco00260; eco01120; eco01230; eco01230	3.698	3.766
Deoxyribose-phosphate aldolase/phospho-2-dehydro-3-deoxyheptonate aldolase	BP; MF	K08321		3.717	4.301

Transaldolase	BP; CC; MF	K00616	eco01200; eco01120; eco01230; eco01230; eco01110; eco00030	3.730	4.175
Transketolase	MF	K00615	eco01200; eco01120; eco01230; eco01230; eco01110; eco00030	3.749	4.933
Protein YebF	CC			3.757	3.581
Intracellular protease, PfpI family	BP; MF	K05520		3.778	4.067
Alcohol dehydrogenase GroES domain protein	MF			3.779	3.682
Conserved protein				3.798	4.182
Disulfide bond reductase, subunit of disulfide reductase	MF	K00799	eco00480	3.823	6.309
Alcohol dehydrogenase zinc-binding domain protein	MF	K13979		3.928	4.147
Conserved protein				3.929	1.756
ABC transporter related	BP; MF	K10010	eco02010	3.933	4.507
GadC GABA APC transporter	CC; MF			3.941	4.593
Probable lipid kinase YegS	BP; CC; MF	K07029		3.972	3.915
OsmF, subunit of YehW/YehX/YehY/YehZ ABC transporter	MF	K05845	eco02010	3.987	3.860
2-deoxy-D-gluconate 3-dehydrogenase, N-ter	BP; MF	K00065	eco00040	4.003	2.179
Catalase	BP; MF	K03781	eco01110; eco00380; eco00630	4.074	5.020
Outer membrane porin protein C (OmpC)	BP; CC; MF	K09475	eco02020	4.085	5.280
A,α-trehalose-phosphate synthase (UDP-forming)	BP; MF	K00697	eco00500; eco01230	4.092	1.670
Putative uncharacterized protein yahO				4.157	4.124
Glucan 1,4-α-maltohexaosidase	BP; MF	K01176	eco00500; eco01230	4.165	4.102
Alcohol dehydrogenase GroES domain protein	MF	K12957		4.169	5.000
Acylphosphatase	MF	K01512	eco00620; eco01120; eco0063	4.192	5.493
dITP/XTP pyrophosphatase	BP; MF	K02428	eco00230; eco01100; eco00240	4.211	4.293
Glutamate decarboxylase	BP; MF	K01580	eco00650; eco00630	4.234	5.266
Isochorismatase hydrolase	MF			4.279	4.896
2-methylisocitrate lyase	BP; MF	K03417	eco00640	4.295	1.980
D-tagatose-1,6-bisphosphate aldolase subunit GatY	BP; MF	K08302	eco00052; eco01100	4.345	4.367
Conserved protein				4.452	5.600
2-methylcitrate dehydratase	BP; MF	K01720	eco00640	4.525	2.546
Acetolactate synthase small subunit	MF	K01653	eco00290; eco01230; eco01230; eco01110; eco01210	4.549	2.947
Glyoxalase/bleomycin resistance protein/dioxygenase	MF	K08234		4.655	2.305
Phenylacetaldehyde dehydrogenase	MF	K00146	eco00360; eco01230; eco00380	4.697	1.756
Oxidoreductase	BP; MF	K00540		4.725	4.945
Alcohol dehydrogenase	MF	K13953	eco00626; eco00350; eco01120; eco00625; eco00010; eco01230; eco01110; eco01220; eco00071	4.746	5.445
CsbD family protein				4.759	6.364
Class B acid phosphatase	CC; MF	K03788	eco00740; eco01230	4.771	4.804

2,5-didehydrogluconate reductase	MF	K06221		4.796	3.092
Autonomous glycyl radical cofactor	MF	K06866		4.803	4.872
Hyperosmotically inducible periplasmic protein		K04065		4.879	5.707
Extracellular solute-binding protein family 3 (FliY)	CC; MF	K02424	eco02010	4.982	5.127
Conserved protein		K05594		5.101	6.597
Superoxide dismutase [Cu-Zn]	BP; MF	K04565		5.130	5.987
Putative uncharacterized protein yegP		K09946		5.205	4.299
Component of SufBCD complex	BP	K09014		5.207	3.872
Malate synthase	BP; MF	K01638	eco01200; eco00620; eco01120; eco01230; eco00630	5.511	2.594
Methylglyoxal synthase	BP; MF	K01734	eco00620	5.623	6.024
L-asparaginase, type II	BP; MF	K01424	eco00250; eco01100; eco01110; eco00460	5.985	4.010
Putative uncharacterized protein ygaM				6.028	6.529
Conserved protein with nucleoside triphosphate hydrolase domain	BP; MF	K07180		6.042	3.460
Cytoplasmic trehalase	BP; CC; MF	K01194	eco00500; eco01230	6.131	5.466
Citrate synthase	BP; CC; MF	K01659	eco00640	7.076	4.103
Outer membrane lipoprotein	CC	K07285		7.162	9.015
Hydrogenase (NiFe) small subunit HydA	CC; MF	K06282	eco01120; eco00633	8.660	9.976
Putative lipoprotein				11.345	8.631
Peptidase M48 Ste24p	BP; CC; MF	K07387		11.876	12.857
Outer membrane pore protein N, non-specific (OmpN)	CC; MF	K14062		19.115	24.950
**Differential proteins that showed different expression pattern in p21-cDNA and p21-ORF**					
Conserved protein				0.626	2.806
**Proteins that only differentially expressed in p21-cDNA**					
UPF0352 protein YejL		K09904		0.424	
MltA-interacting MipA family protein	MF	K07274		0.509	
DNA-directed RNA polymerase subunit omega	BP; CC; MF	K03060	eco03020; eco00230; eco01230; eco00240	0.523	
Maltose operon periplasmic	BP; CC	K05775		0.541	
Na(+)/H(+) antiporter NhaB	BP; CC; MF	K03314		0.542	
Inositol monophosphatase	BP; MF	K01092	eco00562; eco01230; eco00521; eco01110	0.558	
Cell division protein ZapB	BP; CC	K09892		0.572	
Aminotransferase class V	BP; MF	K01766		0.576	
GTP cyclohydrolase-2	BP; MF	K01497	eco00740; eco01120; eco00627	0.592	
Ribosomal RNA large subunit methyltransferase I	CC; MF	K06969		0.592	
2-hydroxyglutaryl-CoA dehydratase D-component				0.595	
NADH dehydrogenase subunit E	MF	K00334	eco00190; eco01100	0.598	
Porphobilinogen deaminase	BP; MF	K01749	eco00860; eco01230; eco01110	0.604	
UPF0301 protein YqgE		K07735		0.605	
HU, DNA-binding transcriptional regulator, α subunit	BP; MF	K05787		0.614	
Iron-sulfur cluster insertion protein ErpA	BP; MF	K15724		0.617	

Cold shock protein-like protein	BP; CC; MF	K03704		0.620	
Isoaspartyl dipeptidase	MF	K01305		0.620	
Thymidylate kinase	BP; MF	K00943	eco01100; eco00240	0.627	
Conserved protein		K09896		0.628	
Cell envelope integrity inner membrane protein TolA	CC; MF	K03646		0.629	
Iron-binding protein IscA	BP; MF	K13628		0.632	
tRNA-dihydrouridine synthase	BP; MF	K05539		0.643	
Predicted periplasmic protein, subunit of The Tol-Pal Cell Envelope Complex, Colicin S4 Transport System and The Colicin A Import System				0.643	
AIG2 family protein				0.650	
RecJ	BP; MF	K07462	eco03440; eco03430; eco03410	0.652	
4Fe-4S ferredoxin iron-sulfur binding domain protein	MF			0.654	
ATP synthase epsilon chain	BP; CC; MF	K02114	eco00190; eco01100	0.657	
MazF toxin of the MazF-MazE toxin-antitoxin system that exhibits ribonuclease activity, subunit of MazE-MazF complex	MF	K07171		0.662	
ATP synthase subunit α	BP; CC; MF	K02111	eco00190; eco01100	0.664	
Maf-like protein YceF	CC	K06287		0.667	
MOSC domain containing protein	BP; MF	K07140		1.510	
Aconitate hydratase	MF	K01681	eco01120; eco01230; eco00020; eco01210; eco01200; eco01230; eco01110; eco00640; eco00630	1.513	
Dyp-type peroxidase family	MF	K07223		1.516	
Aminoacyl-histidine dipeptidase	BP; MF	K01270	eco01100; eco00480	1.539	
Predicted oxidoreductase, NAD(P)-binding	MF			1.543	
ArcA transcriptional dual regulator	BP; CC; MF	K07773	eco02020	1.548	
Aldo/keto reductase	MF			1.550	
Conserved metal-binding protein				1.560	
Putative uncharacterized protein ydgH				1.565	
tRNA modification GTPase MnmE	BP; CC; MF	K03650		1.594	
DNA-binding transcriptional activator	CC; MF	K05804		1.596	
Arginine N-succinyltransferase	BP; MF	K00673	eco00330	1.828	
Conserved protein		K05777		1.892	
3-mercaptopyruvate sulfurtransferase	MF	K01011	eco00920; eco02010	1.954	
Asparagine synthetase	BP; MF	K01953	eco00250; eco01100; eco01110	2.046	
Phosphoribosylformylglycinamidine synthase	BP; CC; MF	K01952	eco00230; eco01100; eco01110	2.585	
Glutamate 5-kinase	BP; CC; MF	K00931	eco00330; eco01230; eco01230	2.689	
Uronate isomerase	BP; MF	K01812	eco01100; eco00040	3.525	
**Proteins that only differentially expressed in p21-ORF**					
Transcription termination factor Rho	BP; MF	K03628	eco03018		0.369
Peptide chain release factor 3	BP; CC; MF	K02837			0.430

ABC transporter related	BP; MF	K06158		0.503
Aminomethyltransferase	BP; CC; MF	K00605	eco01200; eco00260	0.519
Acetyl-coenzyme A synthetase	BP; MF	K01895	eco00680; eco01200; eco00620; eco01120; eco01230	0.520
tRNA (guanine-N(7)-)-methyltransferase	BP; MF	K03439		0.535
Pseudouridine synthase	BP; MF	K06178		0.554
Aconitate hydratase 2	BP; CC; MF	K01682	eco01120; eco01230; eco00030	0.568
CRP transcriptional dual regulator	BP; CC; MF	K10914	eco02020	0.571
DNA-binding response regulator in two-component regulatory system with NarQ or NarX	BP; CC; MF	K07685	eco02020	0.574
Glutamate-cysteine ligase	BP; MF	K01919	eco01100; eco00480	0.575
Soluble pyridine nucleotide transhydrogenase	BP; CC; MF	K00322	eco00760; eco01230	0.590
Mannitol-1-phosphate 5-dehydrogenase	BP; MF	K00009	eco00051	0.596
3-dehydroquinate dehydratase	BP; MF	K03785	eco00400; eco01230; eco01230; eco01110	0.614
Bifunctional protein HldE	BP; MF	K03272	eco01100; eco00540	0.614
Glyoxylate/hydroxypyruvate reductase B	CC; MF	K00090	eco01120; eco01230; eco00020; eco00480; eco01210; eco01200; eco01230; eco01110	0.629
Putative N-acetylmannosamine-6-phosphate 2-epimerase	BP; MF	K01788	eco00520	0.637
50S ribosomal protein L9	BP; CC; MF	K02939	eco03010	0.645
30S ribosomal protein S8	BP; CC; MF	K02994	eco03010	0.650
GTP-binding protein YchF	MF	K06942		0.650
Isocitrate dehydrogenase [NADP]	BP; MF	K00031	eco01120; eco01230; eco00020; eco01210; eco01200; eco01230; eco01110; eco00630	0.655
Chromosome partition protein MukB	BP; CC; MF	K03632		0.660
Uridine phosphorylase	BP; CC; MF	K00757	eco01100; eco00240	1.514
D-aminopropanol dehydrogenase / glycerol dehydrogenase	MF	K00005	eco01100; eco00561	1.571
Glycerophosphoryl diester phosphodiesterase	BP; MF	K01126	eco00564	1.601
Pyruvate kinase	BP; MF	K00873	eco00620; eco01230	1.607
N-acetylglucosamine-6-phosphate deacetylase	BP; MF	K01443	eco00520; eco01110	1.620
Glucosamine-6-phosphate deaminase	BP; MF	K02564	eco00520; eco01110	1.628
Universal stress protein	BP	K14065		1.639
Trp operon repressor	BP; CC; MF	K03720		1.685
Outer membrane lipoprotein	BP; CC; MF	K01051	eco00500; eco01230	1.689
Integration host factor subunit α	BP; MF	K04764		1.732

BP, biological process; CC, cellular component; MF, molecular function.
