# Supplementary material for: Proteomic Analyses Reveal the Mechanism of Dunaliella salina Ds-26-16 Gene Enhancing Salt Tolerance in Escherichia coli
Source: PLoS One. 2016 May 2;11(5):e0153640. doi: 10.1371/journal.pone.0153640 (PMC4852897; doi:10.1371/journal.pone.0153640)
Supplement: S3 Table — (PDF) [file pone.0153640.s009.pdf]

**S3 Table. GO classification of differential proteins identified in p21-cDNA strain**

| GO class | GO Term | No. of Protein |
|---|---|---|
| **Biological Process** | metabolic process | 327 |
| | cellular process | 319 |
| | single-organism process | 83 |
| | localization | 58 |
| | biological regulation | 35 |
| | response to stimulus | 30 |
| | cellular component organization or biogenesis | 27 |
| **Cellular Component** | cell | 213 |
| | membrane | 128 |
| | macromolecular complex | 53 |
| | organelle | 30 |
| **Molecular Function** | catalytic activity | 389 |
| | binding | 292 |
| | transporter activity | 70 |
| | structural molecule activity | 28 |
