# Supplementary material for: Proteomic Analyses Reveal the Mechanism of Dunaliella salina Ds-26-16 Gene Enhancing Salt Tolerance in Escherichia coli
Source: PLoS One. 2016 May 2;11(5):e0153640. doi: 10.1371/journal.pone.0153640 (PMC4852897; doi:10.1371/journal.pone.0153640)
Supplement: S4 Table — (PDF) [file pone.0153640.s010.pdf]

S4 Table. Main differential proteins identified in p21-cDNA strain under salt stress

Protein category	Protein description	KEGG gene	Fold change
**Amino acid and organic acid metabolism**	5-methyltetrahydropteroyltriglutamate-homocysteine methyltransferase	metE; EC: 2.1.1.14	0.215
	Ketol-acid reductoisomerase	ilvC; EC: 1.1.1.86	0.255
	Acetylglutamate kinase	argB; EC: 2.7.2.8	0.256
	Acetylornithine/succinyldiaminopimelate aminotransferase	argD; EC: 2.6.1.11 2.6.1.17	0.270
	Argininosuccinate lyase	argH; EC: 4.3.2.1	0.286
	Lysine decarboxylase	EC:4.1.1.18	0.289
	Carbamoyl-phosphate synthase small chain	carA; EC: 6.3.5.5	0.297
	Carbamoyl-phosphate synthase large chain	carB; EC: 6.3.5.5	0.304
	2-hydroxy-3-oxopropionate reductase	EC: 1.1.1.60	0.315
	Bifunctional dihydroneopterin aldolase/dihydroneopterin triphosphate 2'-epimerase	folB; EC: 4.1.2.25	0.325
	N-succinylglutamate 5-semialdehyde dehydrogenase	astD; EC: 1.2.1.71	0.327
	Ornithine carbamoyltransferase	OTC; EC: 2.1.3.3	0.330
	Aspartate carbamoyltransferase	pyrB; EC: 2.1.3.2	0.338
	Phosphoenolpyruvate synthase	pps; EC: 2.7.9.2	0.343
	Dihydroxy-acid dehydratase	ilvD; EC: 4.2.1.9	0.344
	Aspartokinase	lysC; EC: 2.7.2.4	0.346
	N-acetyl-gamma-glutamyl-phosphate reductase	argC; EC: 1.2.1.38	0.375
	N-acetylneuraminate lyase	EC: 4.1.3.3	0.387
	L-lactate dehydrogenase [cytochrome]	EC: 1.1.2.3	0.400
	3-oxoacyl-[acyl-carrier-protein] synthase 2	fabF; EC: 2.3.1.179	0.416
	Aspartate-semialdehyde dehydrogenase		0.439
	GTP cyclohydrolase 1	gch1; EC: 3.5.4.16	0.467
	Glycerate kinase I	glxK; EC: 2.7.1.31	0.484
	3-phosphoserine phosphatase	serB; EC: 3.1.3.3	0.508
	Cysteine-tRNA ligase	carS; EC: 6.1.1.16	0.514
	Aromatic-amino-acid transaminase	tyrB; EC: 2.6.1.57	0.514
	7,8-dihydropteroate synthase	folP; EC: 2.5.1.15	0.518
	D-serine dehydratase	EC: 4.3.1.18	0.529
	2,3,4,5-tetrahydropyridine-2,6-dicarboxylate N-succinyltransferase	dapD; EC: 2.3.1.117	0.535
	Aspartate kinase	thrA; EC: 2.7.2.4 1.1.1.3	0.540
	PabB, subunit of aminodeoxychorismate synthase and para-aminobenzoate synthase multi-enzyme complex	pabB; EC: 2.6.1.85	0.575
	Acetylornithine deacetylase	argE; EC: 3.5.1.16	0.589
	Bifunctional protein FolC	folC; EC: 6.3.2.12 6.3.2.17	0.599
	Ornithine decarboxylase, biosynthetic	EC: 4.1.1.17	0.601
	N-succinylarginine dihydrolase	astB; EC: 3.5.3.23	1.592

	Asparaginase II	EC: 3.5.1.1	1.766
	Arginine N-succinyltransferase	astA; EC: 2.3.1.109	1.828
	Asparagine synthetase	asnB; EC: 6.3.5.4	2.046
	3-isopropylmalate dehydratase small subunit	leuD; EC: 4.2.1.33 4.2.1.35	2.209
	Nitrate reductase 2 (NRZ), α subunit	narG; EC: 1.7.99.4	2.301
	Chorismate mutase	pheA; EC: 5.4.99.5 4.2.1.51	2.467
	Phosphoribosylformylglycinamidine synthase	purL; EC: 6.3.5.3	2.585
	Dihydrofolate reductase/dihydromonapterin reductase	folM; EC: 1.5.1.- 1.5.1.3	2.621
	Isocitrate lyase	EC: 4.1.3.1	2.623
	Glutamate 5-kinase	proB; EC: 2.7.2.11	2.689
	Cysteine desulfurase	sufS; EC: 2.8.1.7 4.4.1.16	2.716
	AsnC transcriptional dual regulator	asnC	2.740
	Nitrate reductase 1, α subunit	narG; EC: 1.7.99.4	2.744
	Nitrate reductase 2 (NRZ), β subunit	narH; EC: 1.7.99.4	2.811
	Gamma-glutamyltransferase	ggt; EC: 2.3.2.2	2.954
	4-aminobutyrate aminotransferase	gabT; EC: 2.6.1.19 2.6.1.22	2.977
	D-cysteine desulfhydrase	dcyD; EC: 4.4.1.15	3.032
	Succinate semialdehyde dehydrogenase, $NADP^+$-dependent	gabD; EC: 1.2.1.16 1.2.1.79 1.2.1.20	3.141
	Succinylornithine transaminase	astC; EC: 2.6.1.81	3.220
	Glutaminase	glsA; EC: 3.5.1.2	3.626
	Phosphoadenosine phosphosulfate reductase	cysH; EC: 1.8.4.8	3.667
	Glutamate decarboxylase	EC: 4.1.1.15	4.113
	2-methylisocitrate lyase	prpB; EC: 4.1.3.30	4.295
	2-methylcitrate dehydratase	prpD; EC: 4.2.1.79	4.525
	L-asparaginase, type II	EC: 3.5.1.1	5.985
**Carbohydrate metabolism**	2-dehydro-3-deoxyphosphooctonate aldolase	kdsA; EC: 2.5.1.55	0.203
	Ybl99	rfbC; EC: 5.1.3.13	0.205
	Nucleotide sugar dehydrogenase	UGDH; EC: 1.1.1.22	0.227
	Bifunctional polymyxin resistance protein ArnA	arnA; EC: 2.1.2.13 1.1.1.305	0.231
	Lipopolysaccharide core heptose(II)-phosphate phosphatase		0.284
	L-rhamnose isomerase	rhaA; EC: 5.3.1.14	0.289
	UDP-4-amino-4-deoxy-L-arabinose-oxoglutarate aminotransferase	arnB; EC: 2.6.1.87	0.299
	(D)-galactarate dehydrogenase	garD; EC: 4.2.1.42	0.313
	Undecaprenyl-phosphate 4-deoxy-4-formamido-L-arabinose transferase	arnC; EC: 2.4.2.53	0.327
	Cytoplasmic glycerophosphodiester phosphodiesterase	EC: 3.1.4.46	0.358
	L-fucose isomerase	fucI; EC: 5.3.1.25	0.366
	D-tagatose-1,6-bisphosphate aldolase subunit GatZ	gatZ-kbaZ	0.382
	5-keto-4-deoxy-D-glucarate aldolase	garL; EC: 4.1.2.20	0.385

	Phosphorylase	EC: 2.4.1.1	0.438
	Inositol monophosphatase	EC: 3.1.3.25	0.558
	Dihydrolipoamide acetyltransferase	dlaT; EC: 2.3.1.12	0.561
	6-phosphogluconate dehydratase	edd; EC: 4.2.1.12	0.562
	Glycerol kinase	EC: 2.7.1.30	1.888
	Periplasmic trehalase	EC: 3.2.1.28	2.213
	Deoxyribose-phosphate aldolase	deoC; EC: 4.1.2.4	2.285
	L-1,2-propanediol oxidoreductase	fucO; EC: 1.1.1.77	2.550
	4-deoxy-L-threo-5-hexosulose-uronate ketol-isomerase	kduI; EC: 5.3.1.17	2.730
	Glycerol-3-phosphate dehydrogenase (Anaerobic), large subunit, subunit of glycerol-3-phosphate-dehydrogenase, anaerobic	glpA; EC: 1.1.5.3	3.202
	Anaerobic glycerol-3-phosphate dehydrogenase subunit B	glpB; EC: 1.1.5.3	3.239
	Uronate isomerase	uxaC; EC: 5.3.1.12	3.525
	Fructose bisphosphate aldolase monomer, subunit of fructose bisphosphate aldolase class I	fbaB; EC: 4.1.2.13	3.698
	Transaldolase	EC: 2.2.1.2	3.730
	α,α-trehalose-phosphate synthase (UDP-forming)	otsA; EC: 2.4.1.15	4.092
	Glucan 1,4-α-maltohexaosidase	EC: 3.2.1.1	4.165
	D-tagatose-1,6-bisphosphate aldolase subunit GatY	gatY; EC: 4.1.2.40	4.345
	Methylglyoxal synthase	EC: 4.2.3.3	5.623
	Cytoplasmic trehalase	EC: 3.2.1.28	6.131
	Citrate synthase	prpC; EC: 2.3.3.5	7.076
**Energy metabolism**	Cytochrome bo terminal oxidase subunit II, subunit of cytochrome bo terminal oxidase	cyoA; EC: 1.10.3.-	0.269
	Cytochrome bo terminal oxidase subunit I, subunit of cytochrome bo terminal oxidase	cyoB; EC: 1.10.3.-	0.350
	NADH:ubiquinone oxidoreductase II	ndh; EC: 1.6.99.3	0.382
	S-adenosylmethionine synthase	metK; EC: 2.5.1.6	0.400
	NADH-quinone oxidoreductase, F subunit	nuoF; EC: 1.6.5.3	0.453
	Cytochrome bd ubiquinol oxidase subunit I	cydA; EC: 1.10.3.-	0.470
	NADH-quinone oxidoreductase subunit N	nuoN; EC: 1.6.5.3	0.480
	Aspartate ammonia-lyase	aspA; EC: 4.3.1.1	0.485
	NADH-quinone oxidoreductase, chain G	nuoG; EC: 1.6.5.3	0.485
	2-oxoglutarate dehydrogenase, E1 subunit	ogdH; EC: 1.2.4.2	0.518
	NADH-quinone oxidoreductase subunit I	nuoI; EC: 1.6.5.3	0.54
	Cytochrome bd-I terminal oxidase subunit II, subunit of cytochrome bd-I terminal oxidase	cydB; EC: 1.10.3.-	0.543
	Succinate dehydrogenase and fumarate reductase iron-sulfur protein	sdhB; EC: 1.3.99.1	0.544
	Succinate dehydrogenase cytochrome b556 small membrane subunit	sdhD; EC: 1.3.5.1 1.3.5.4	0.555
	2-oxoglutarate dehydrogenase, E2 subunit, dihydrolipoamide succinyltransferase	dlsT; EC: 2.3.1.61	0.579
	NADH dehydrogenase subunit E	nuoE; EC: 1.6.5.3	0.598
	NADH-quinone oxidoreductase subunit K	nuoK; EC: 1.6.5.3	0.615
	ArcA transcriptional dual regulator	arcA	1.548
	1,4-α-glucan branching enzyme GlgB	glgB; EC: 2.4.1.18	1.665

	Fumarate reductase (Anaerobic), Fe-S subunit	frdB; EC: 1.3.99.1	1.670
	Glucose-1-phosphate adenylyltransferase	glgC; EC: 2.7.7.27	1.681
	Malate synthase	EC:2.3.3.9	5.511
**Oxidative stress protection**	Superoxide dismutase		1.563
	Glutathione transferase		1.744
	Catalase-peroxidase	katG; EC: 1.11.1.21	1.816
	Glutathione S-transferase	gst; EC: 2.5.1.18	1.855
	Glutathione S-transferase domain protein		1.902
	Exoribonuclease 2		2.253
	HTH-type transcriptional regulator IscR	iscR	2.332
	Cold shock protein E		2.890
	Lon protease		3.074
	Glutaredoxin 2 (Grx2)	Grx2	3.443
	Glutathione peroxidase	EC: 1.11.1.9	3.572
	Osmotically inducible peroxidase OsmC	osmC	3.660
	Catalase	katE; EC: 1.11.1.6	4.074
	Superoxide dismutase [Cu-Zn]		5.130
**Membrane proteins and ABC transporters**	C4-dicarboxylate transport protein	dctA	0.228
	Fused trehalose(Maltose)-specific PTS enzyme: IIB component/IIC component	PTS	0.250
	OmpA/MotB domain protein	OmpA	0.261
	Arginine transporter subunit	artJ	0.277
	Copper/silver efflux system, membrane fusion protein	cusB	0.277
	Galactitol-specific enzyme IIC component of PTS	PTS	0.289
	Outer membrane channel protein	tolC	0.294
	FepA, outer membrane receptor for ferric enterobactin (Enterochelin) and colicins B and D, subunit of Outer Membrane Ferric Enterobactin Transport System and Ferric Enterobactin Transport System	FepA	0.315
	Galactitol-specific enzyme IIB component of PTS	PTS; EC: 2.7.1.69	0.349
	OmpF, subunit of outer membrane porin F and The Colicin A Import System	ompF	0.378
	Cationic amino acid ABC transporter, periplasmic binding protein	hisJ	0.390
	Protein translocase subunit SecD	secD	0.411
	LPS-assembly protein LptD	lptD	0.415
	NADH-quinone oxidoreductase subunit A	nuoA; EC: 1.6.5.3	0.426
	NADH-quinone oxidoreductase subunit B	nuoB; EC: 1.6.5.3	0.429
	Oligopeptide transporter ATP-binding component	oppD	0.438
	ABC transporter, CydDC cysteine exporter (CydDC-E) family, permease/ATP-binding protein CydD	cydD	0.451
	Membrane protein insertase YidC	yidC	0.454
	Outer membrane protein X	OmpX	0.460
	ABC transporter, CydDC cysteine exporter (CydDC-E) family, permease/ATP-binding protein CydC	cydC	0.466
	Maltoporin		0.467
	Protein-export membrane protein SecF	secF	0.467

	Lipoprotein releasing system, transmembrane protein, LolC/E family	lolC/E	0.474
	Outer membrane phospholipase A	pldA; EC: 3.1.1.32 3.1.1.4	0.475
	ABC transporter related	malK; EC: 3.6.3.-	0.475
	Binding-protein-dependent transport systems inner membrane component	malF	0.480
	NADH-quinone oxidoreductase subunit C/D	nuoCD; EC: 1.6.5.3	0.499
	ATP synthase subunit b	epsilon; EC: 3.6.3.14	0.510
	LPS-assembly lipoprotein LptE	lptE	0.521
	Oligopeptide transporter subunit	oppA	0.523
	Sec-independent protein translocase protein TatA	tatA	0.523
	Cell division protein FtsX	ftsX	0.540
	Maltose operon periplasmic		0.541
	Na(+)/H(+) antiporter NhaB	nhaB	0.542
	Outer membrane protein assembly factor BamA	bamA	0.565
	Outer membrane protein assembly factor BamD	bamD	0.575
	Outer membrane protein assembly factor BamB	bamB	0.579
	Outer-membrane lipoprotein LolB	lolB	0.583
	ATP synthase epsilon chain	EC: 3.6.3.14	0.657
	ATP synthase subunit α	atpf1A(a); EC: 3.6.3.14	0.664
	LYSR-type transcriptional regulator	LYSR	1.474
	Phosphate-binding protein PstS	pstS	1.524
	ArgT, subunit of lysine/arginine/ornithine ABC Transporter	argT	1.552
	Extracellular solute-binding protein family 1	potD	1.638
	AraF, subunit of arabinose ABC transporter	araF	1.782
	MlaB, subunit of phospholipid ABC transporter	mlaB	1.867
	ChbB, subunit of EIIChb	EC: 2.7.1.69	1.884
	DNA-binding transcriptional activator		1.895
	ABC transporter related	rbsA; EC: 3.6.3.17	2.221
	Glycerol-3-phosphate dehydrogenase (Anaerobic), small subunit, subunit of glycerol-3-phosphate-dehydrogenase, anaerobic	glpC; EC: 1.1.5.3	2.269
	Nickel ABC transporter, periplasmic nickel-binding protein	nikA	2.532
	D-xylose ABC transporter, periplasmic substrate-binding protein	xylF	2.583
	D-ribose transporter subunit	rbsB	2.637
	Extracellular solute-binding protein family 1	PotF	2.654
	Cysteine desulfurase ATPase component		2.887
	ABC-type sugar transport system periplasmic component-like protein	lsrB	2.967
	Bacterioferritin		3.130
	Efflux transporter, RND family, MFP subunit		3.368
	Lipopolysaccharide transport periplasmic protein LptA	lptA	3.403
	OsmF, subunit of YehW/YehX/YehY/YehZ ABC transporter	opuC	3.987
	Outer membrane porin protein C (OmpC)	ompC	4.085

	Extracellular solute-binding protein family 3 (FliY)	fliY	4.982
	Outer membrane lipoprotein		7.162
	Outer membrane pore protein N, non-specific (OmpN)	ompN	19.115
**Peptidoglycan biosynthesis**	Fused penicillin-binding protein 1a: murein transglycosylase/murein transpeptidase	mrcA; EC: 2.4.1.- 3.4.-.-	0.375
	UDP-N-acetylenolpyruvoylglucosamine reductase	murB; EC: 1.3.1.98	0.431
	UDP-N-acetylmuramoyl-tripeptide-D-alanyl-D-alanine ligase	murF; EC: 6.3.2.10	0.487
	UDP-N-acetylmuramoyl-L-alanyl-D-glutamate-2,6-diaminopimelate ligase	murE; EC: 6.3.2.13	0.494
	UDP-N-acetylglucosamine-N-acetylmuramyl-(pentapeptide) pyrophosphoryl-undecaprenol N-acetylglucosamine transferase	murG; EC: 2.4.1.227	0.522
	UDP-N-acetylglucosamine 1-carboxyvinyltransferase	murA; EC: 2.5.1.7	0.568
	Glutamine-fructose-6-phosphate aminotransferase [isomerizing]	EC: 2.6.1.16	0.596
	D-alanine-D-alanine ligase	ddl; EC: 6.3.2.4	0.604
	BolA transcriptional dual regulator	bolA	2.284
